# Reprogramming of plants during systemic acquired resistance

**DOI:** 10.3389/fpls.2013.00252

**Published:** 2013-07-15

**Authors:** Katrin Gruner, Thomas Griebel, Hana Návarová, Elham Attaran, Jürgen Zeier

**Affiliations:** ^1^Department of Biology, Heinrich Heine UniversityDüsseldorf, Germany; ^2^Department of Plant-Microbe Interactions, Max Planck Institute for Plant Breeding ResearchCologne, Germany; ^3^Department of Plant Biology, Michigan State UniversityEast Lansing, MI, USA

**Keywords:** systemic acquired resistance, transcriptional profiling, salicylic acid, gene classification, gene regulation, defense priming

## Abstract

Genome-wide microarray analyses revealed that during biological activation of systemic acquired resistance (SAR) in Arabidopsis, the transcript levels of several hundred plant genes were consistently up- (SAR^+^ genes) or down-regulated (SAR^−^ genes) in systemic, non-inoculated leaf tissue. This transcriptional reprogramming fully depended on the SAR regulator FLAVIN-DEPENDENT MONOOXYGENASE1 (FMO1). Functional gene categorization showed that genes associated with salicylic acid (SA)-associated defenses, signal transduction, transport, and the secretory machinery are overrepresented in the group of SAR^+^ genes, and that the group of SAR^−^ genes is enriched in genes activated via the jasmonate (JA)/ethylene (ET)-defense pathway, as well as in genes associated with cell wall remodeling and biosynthesis of constitutively produced secondary metabolites. This suggests that SAR-induced plants reallocate part of their physiological activity from vegetative growth towards SA-related defense activation. Alignment of the SAR expression data with other microarray information allowed us to define three clusters of SAR^+^ genes. Cluster I consists of genes tightly regulated by SA. Cluster II genes can be expressed independently of SA, and this group is moderately enriched in H_2_O_2_- and abscisic acid (ABA)-responsive genes. The expression of the cluster III SAR^+^ genes is partly SA-dependent. We propose that SA-independent signaling events in early stages of SAR activation enable the biosynthesis of SA and thus initiate SA-dependent SAR signaling. Both SA-independent and SA-dependent events tightly co-operate to realize SAR. SAR^+^ genes function in the establishment of diverse resistance layers, in the direct execution of resistance against different (hemi-)biotrophic pathogen types, in suppression of the JA- and ABA-signaling pathways, in redox homeostasis, and in the containment of defense response activation. Our data further indicated that SAR-associated defense priming can be realized by partial pre-activation of particular defense pathways.

## Introduction

Plants are equipped with a multi-layered immune system that employs constitutive and inducible defense strategies to antagonize colonization by pathogenic microbes (Spoel and Dong, 2012). In pathogen-inoculated plant tissue, conserved microbial structures (pathogen-associated molecular patterns, PAMPs) elicit PAMP-triggered immunity (PTI), a basal resistance response that contains the extent of infection by compatible pathogen isolates. Following recognition of race-specific pathogen effectors by plant immune receptors (“resistance proteins”), plants are able to activate effector-triggered immunity (ETI) that usually results in a hypersensitive response (HR) at inoculation sites and provides effective local resistance to pathogens with a biotrophic or hemibiotrophic lifestyle (Jones and Dangl, 2006). Although ETI is associated with stronger local responses than PTI, the signaling networks underlying both resistance forms partially overlap (Tsuda et al., 2009).

A localized microbial infection of a single or a few leaves can also immunize the rest of the foliage to subsequent infection, a phenomenon known as systemic acquired resistance (SAR) (Fu and Dong, 2013; Shah and Zeier, 2013). Once activated, SAR provides enhanced resistance to a broad range of (hemi) biotrophic fungal, bacterial, and viral pathogens (Sticher et al., 1997). The SAR response is initiated by microbes eliciting PTI or ETI at inoculation sites, and can also be triggered by localized leaf treatment with purified PAMPs. The mechanistic principles leading to SAR induction by different types of bacterial pathogens and the resulting systemic immunization patterns are highly overlapping (Mishina and Zeier, 2007; Jing et al., 2011). For instance, compatible, PTI-inducing *Pseudomonas syringae* pv. *maculicola* ES4326 (*Psm*) and ETI-inducing *Psm avrRpm1* elicit highly similar systemic responses in *Arabidopsis thaliana* (Arabidopsis) plants, including systemic accumulation of the SAR immune signals pipecolic acid (Pip) and salicylic acid (SA), and enhanced systemic expression of a variety of classical SAR marker genes such as *PATHOGENESIS-RELATED GENE 1 (PR1), PR2*, and *PR5* (Mishina and Zeier, 2006; Návarová et al., 2012).

Effective long-distance communication between inoculated (1°) leaves and distant (2°, “systemic”) leaves is required for the activation of SAR. Several plant-derived substances have been proposed to participate in SAR long-distance signaling (Dempsey and Klessig, 2012; Shah and Zeier, 2013). These involve the putative lipid transfer protein DEFECTIVE IN INDUCED RESISTANCE1 (DIR1), the methyl ester of SA (MeSA), glycerol-3-phosphate (G3P), the diterpenoid dehydroabietinal, the dicarboxylic acid azelaic acid, and the Lys catabolite Pip (Maldonado et al., 2002; Park et al., 2007; Jung et al., 2009; Chanda et al., 2011; Chaturvedi et al., 2012; Návarová et al., 2012). The importance of several of these candidate signals for SAR induction in plants appears to depend on external parameters such as the light environment (Attaran et al., 2009; Liu et al., 2011; Návarová et al., 2012).

At the onset of SAR, the long-distance information released from 1° leaves is supposed to be perceived in 2° leaf tissue (Shah and Zeier, 2013), and a feedback amplification mechanism in 2° leaves that involves Pip and SA then ensures the activation of SAR (Návarová et al., 2012). The Lys aminotransferase AGD2-LIKE DEFENSE RESPONSE PROTEIN1 (ALD1), whose expression is intensified in 1 and 2° leaves during SAR, is required for SAR activation (Song et al., 2004a,b). ALD1 generates the non-protein amino acid Pip, which has recently been identified as a critical metabolic SAR signal (Návarová et al., 2012). Pip-mediated resistance requires FLAVIN-DEPENDENT MONOOXYGENASE1 (FMO1), another indispensable SAR module (Mishina and Zeier, 2006; Návarová et al., 2012). Pip enhances both its own biosynthesis and downstream signaling in SAR via intensification of *ALD1* and *FMO1* expression, and systemic accumulation of the amino acid is required for the de novo synthesis of SA in 2° leaf tissue (Návarová et al., 2012). SA is a second critical SAR metabolite that is produced in plants from chorismate by ISOCHORISMATE SYNTHASE1 (ICS1) (Wildermuth et al., 2001; Métraux, 2002). SA induces SAR-related gene expression via the downstream regulator NON-EXPRESSER OF PR GENES1 (NPR1), a transcriptional co-activator and bona fide SA receptor (Wu et al., 2012; Fu and Dong, 2013). SAR-induced plants therefore exhibit increased expression of a number of *PR* genes which is presumed to directly contribute to their state of increased disease resistance (Sticher et al., 1997). Additionally, SAR confers defense priming, which enables plants to more effectively respond to future pathogen encounter (Jung et al., 2009; Návarová et al., 2012).

The interaction between Arabidopsis and *P. syringae* represents a useful model system to elucidate the molecular principles underlying inducible plant immunity (Katagiri et al., 2002). Large scale transcriptional profiling in Arabidopsis has been used to better understand PTI- and ETI-associated defense networks that are activated at sites of bacterial inoculation, and the mode of action of bacterial effectors to promote disease (Thilmony et al., 2006; Truman et al., 2006; Wang et al., 2008; Tsuda et al., 2009). Moreover, genome-wide microarray analyses have been employed to characterize Arabidopsis transcriptional responses induced by the synthetic resistance activator S-methyl-1,2,3-benzothiadiazole-7-carbothioate (BTH), which is often considered as a functional SA analogue (Wang et al., 2006). A DNA microarray representing about 25–30 % of the Arabidopsis genes has also been used to monitor and analyse gene expression changes under different SAR-inducing conditions (Maleck et al., 2000).

In the current study, we aimed to characterize biologically-induced SAR in Arabidopsis at the whole genome level, classify SAR-regulated genes according to their function and regulation of expression, derive molecular and physiological characteristics of the SAR-induced state, and further clarify the role of *FMO1* in SAR. We therefore analysed the transcriptional changes that occur in upper 2° leaf tissue upon SAR induction with *Psm* bacteria in lower 1° leaves by use of ATH1 microarray chip analyses in the Arabidopsis wild-type and *fmo1* mutant plants. These analyses revealed that SAR is associated with massive transcriptional reprogramming in systemic tissue that virtually fully depends on *FMO1*. Alignment of the SAR expression data with publicly available microarray information from defense-, stress-, and hormone-related experiments allowed us to obtain information about the regulation of genes that are up- and down-regulated during SAR. For instance, within the group of genes up-regulated during SAR, subgroups consisting of SA-independent, SA-dependent, and partially SA-dependent genes could be discriminated. Moreover, our evaluation indicated that overlapping and contrasting regulatory principles exist for the induction of local resistance responses and SAR. Further, functional categorization of SAR-related genes suggested that, upon SAR induction, plants redirect some of their resources from vegetative growth towards defense.

## Materials and methods

### Plant material and growth conditions

*Arabidopsis thaliana* (L.) Heynh. ecotype Col-0 and *fmo1* mutant plants (T-DNA insertion line SALK_026163; Mishina and Zeier, 2006) were grown in individual pots on an autoclaved mixture of soil (Klasmann, Beetpflanzensubstrat Typ R.H.P.16), vermiculite and sand (10:0.5:0.5) in a controlled environmental chamber (J-66LQ4, Percival, Boone, IA) within 9 h day (9 AM to 6 PM; photon flux density 70 μmol m^−2^ s^−1^) and 15 h night periods in a relative humidity of 70%. Growth temperatures during the day and night period were set to 21°C and 18°C, respectively. SAR experiments were performed with 5-6 week-old plants exhibiting a uniform appearance.

### SAR experiments

Overnight cultures of *Pseudomonas syringae* pv. *maculicola* ES4326 (*Psm*) were cultivated as described (Mishina and Zeier, 2006). For SAR induction, plants were infiltrated between 10 AM and 11 AM into three lower (1°) leaves (typically leaf 7–9) with a suspension of *Psm* in 10 mM MgCl_2_ [optical density at 600 nm (OD_600_) = 0.005]. Infiltration with 10 mM MgCl_2_ served as the mock-control treatment. Upper (2°) leaves (typically leaf 10–12) were harvested and shock-frozen in liquid nitrogen two days after the treatment of 1° leaves. Together, three biologically independent SAR experiments were performed for microarray analyses (see below), and two further biological replicates were performed for qPCR-based expression analyses of selected genes (Table 2).

### RNA isolation

Total RNA was isolated from frozen leaves using QIAzol® Lysis Reagent (Qiagen, http://www.qiagen.com/) following the manufacturer's instructions. For each sample, two leaves from different plants that received the same treatment were used.

### Quantitative real-time PCR analysis

RNA samples were reverse-transcribed with the Omniscript RT Kit (Qiagen) using 1 μg of total RNA. 2.5 μl of cDNA and 5 μl of SensiFAST™ SYBR No-ROX Kit (Bioline) were used in PCR reactions of 10 μl total reaction volume containing 0.75 μM gene-specific primers. The At4g26410 gene which is non-responsive to *P. syringae*-inoculation was used as a reference gene (Czechowski et al., 2005). The following primers were used to obtain the expression data for SAR experiments 4 and 5 (Table 2): *PR1-FORWARD*: 5′-GTGCTCTTGTTCTTCCCTCG-3′, *PR1-REVERSE*: 5′-GCCTGGTTGTGAACCCTTAG-3′, *PR2-FW*: 5′-TCAAGGAAGGTTCAGGGATG-3′, *PR2-RV*: 5′-GAGATTCACGAGCAAGGGAG-3′, *PR5-FW*: 5′-ATCGGGAGATTGCAAATACG-3′, *PR5-RV*: 5′-ATGACCTTAAGCATGTCGGG-3′, *AGP5-FW*: 5′-CTACTGAATCTCCACCAGCTC-3′, *AGP5-RV*: 5′-GAGGGAGACTCTGCTAACTG-3′, *CALM3-FW*: 5′-GACTGATGATAAATCGTTGGAG-3′, *CALM3-RV*: 5′-CCCAACAAACTAAGCATCCT-3′, *LTPa-FW*: 5′-GGTTCTACTTCTGACTCTCC-3′, *LTPa-RV*: 5′-GTCCGTCTCCTTCTCCT-3′, *PBS3-FW*: 5′-TGCCTGCTCGAGTCGCAACC-3′, *PBS3-RV*: 5′-TGGACTAAGCCACAGAGCAAATGGC-3′, *UGT76B1-FW*: 5′-CTTTACAAGAGACTAAGGCAG-3′, *UGT76B1-RV*: 5′-CACACCTATCTGTAACTTATCCC-3′, *2OGD1-FW*: 5′-ACCAAATGCAGGTCATAAGC-3′, *2OGD1-RV*: 5′-TGAAGGGAAATAGAAAGTCGG-3′, *NIMIN-1-FW*: 5′-AGTAAGAGAAGACGAAGAAGAG-3′, *NIMIN-1-RV*: 5′-TCCGCCGTTAGATTTCCT-3′, At4g26410-*FW*: 5′-GAGCTGAAGTGGCTTCCATGAC-3′, At4g26410-*RV*: 5′-GGTCCGACATACCCATGACC-3′. The qPCR reaction was performed in triplicate in a Rotor-Gene Q apparatus (Qiagen) using the cycling program: 95°C for 2 min, followed by 40 cycles at 95°C for 7 s, 60°C for 25 s, and finally 72°C for 3 min. The data was analyzed using the Rotor-Gene Q 2.0.2 software, setting the threshold of the normalized fluorescence to 0.1, which corresponded to the exponential phase of the fluorescence signal. The resulting C_T_ and E values were used to calculate the relative mRNA abundance according to the ΔΔC_T_ method (Livak and Schmittgen, 2001). The values were normalized to those of the reference gene and expressed relative to the mock-control sample.

### Microarray analysis and data evaluation

For each SAR microarray experiment, RNA samples from at least 7 replicates for a particular condition (Col-0/mock, Col-0/SAR, *fmo1*/mock, *fmo1*/SAR) were mixed. The pooled RNA samples were quality-checked and expression profiling performed with the GeneChip® Arabidopsis ATH1 Genome Array using the 3′ IVT express kit (Affymetrix) under accreditation conditions (DNAVision, Charleroi, Belgium). The quality of the used GeneChips was assessed and all the samples were hybridized, processed, and scanned in parallel, ensuring that samples could be directly compared to each other. Moreover, the raw microarray data was normalized using the RMA algorithm (Irizarry et al., 2003a,b).

Together, three biologically independent SAR microarray experiments were performed. Statistical analyses of the normalized expression values of the three biological replicates were performed using a two-sided Student's *t*-test. The large scale evaluation of the microarray data was performed using Microsoft Excel® data sheets. Ratios of normalized expression values for *Psm*- and mock samples were calculated ([*P*/M]_SAR_), and genes were arranged and grouped according to the size of their averaged [*P*/M]_SAR_ values with the Excel® data sort function. Similarly, mean gene expression values gathered from other publicly available microarray experiments were determined for each gene, and stimulus-to-mock ratios ([*S*/M]_stimulus_) and ratios of local expression values in Col-0 and mutant leaves following *Psm* inoculation ([Col/*mutant*]_Psm_) were calculated thereof (Table 6). The [*P*/M]_SAR_ ratios for each gene were aligned with the corresponding [S/M]_stimulus_ and [Col/*mutant*]_Psm_ ratios using the “merge” function of FIRe, an Excel® macro designed for rapid microarray data analysis (Garcion et al., 2006). The detailed selection criteria for the categorization of genes are described in the main text. The Arabidopsis Information Resource (TAIR) functional categorization tool was used to classify the genes according to Gene Ontology (GO) descriptions (http://www.arabidopsis.org/tools/bulk/go/index.jsp). The SAR microarray data were deposited in the NASCArrays database (NASCARRAYS-703).

### Determination of camalexin, abscisic acid, and jasmonic acid levels

For the time course analyses of the local and systemic levels of camalexin, JA, and ABA (Figure 8), three 1° leaves of Arabidopsis Col-0 plants were treated with *Psm* (OD_600_ = 0.005) or 10 mM MgCl_2_. At the indicated times after treatment, the treated (1°) leaves and three upper, non-treated (2°) leaves were separately harvested and frozen in liquid nitrogen. Individual samples consisted of 6 leaves from two plants. The determination of the leaf metabolite levels was performed by vapor-phase extraction and subsequent GC/MS analysis as described by Návarová et al. (2012). For quantification, metabolite peaks originating from selected ion chromatograms were integrated: camalexin (m/z 200), ABA (m/z 190), and JA (m/z 224). The area of a substance peak was related to the peak area of dihydrojasmonic acid (m/z 156) or indole-3-propionic acid (m/z 130) for internal standardization. Experimentally determined correction factors were considered.

## Results and discussion

### Functional characterization of the SAR state

The bacterial phytopathogen *Pseudomonas syringae* pv. *maculicola* ES4326 (*Psm*) triggers a classical and robust SAR response in vegetatively growing *Arabidopsis thaliana* plants (Mishina and Zeier, 2006; Jing et al., 2011). SAR induced in Arabidopsis by compatible *Psm* or HR-inducing *Psm avrRpm1* develops between day 1 and day 2 in the whole foliage after a localized leaf inoculation has occurred, and the SAR response is fully established 2 days post inoculation (dpi) (Mishina et al., 2008). We aimed to broaden our understanding of the nature of the SAR state by gathering and analyzing the transcriptional changes that occur upon SAR establishment on the whole Arabidopsis genome level. Therefore, we inoculated lower (1°) rosette leaves of 5 week-old Arabidopsis vegetatively growing Col-0 plants with a suspension of *Psm* (OD_600_ = 0.005) and harvested upper, non-treated (2°) leaves two days after inoculation for RNA extraction. A mock-infiltration of 1° leaves with 10 mM MgCl_2_ served as a control treatment. Affymetrix expression analysis (GeneChip® Arabidopsis ATH1 Genome Array) was then used to compare the expression profiles in 2° leaves of mock- and *Psm*-treated plants. Considering the above-mentioned kinetics of SAR induction in the *Psm*-Col-0 pathosystem, the selected time point at 2 dpi allowed us to characterize the transcriptional reprogramming events when SAR has just fully established, but potentially not the earliest transcriptional events at the very onset of the response. We also included the *fmo1* mutant into the study which is fully compromised in SAR, systemic accumulation of SA, and systemic expression of characteristic SAR marker genes (Mishina and Zeier, 2006).

In sum, we performed three independent SAR experiments that were conducted in the same growth chamber under identical light, temperature and humidity settings at different time periods with distinct batches of plants and pathogens. Each of the independent SAR experiments yielded one pooled RNA sample for both the mock-control and the SAR-induced state that was used for microarray analysis. The pooled RNA samples were derived from at least seven biological replicates within each SAR experiment and every biological replicate consisted of two 2° leaves from distinct plants. Thus, the gene expression samples resulting from an individual SAR experiment exhibited a high intrinsic statistical validity. The 12 pooled RNA samples for microarray analysis (3 Col-0/mock, 3 Col-0/SAR, 3 *fmo1*/mock, 3 *fmo1*/SAR) were quality-checked and expression profiling performed using the 3′ IVT express kit (Affymetrix) under accreditation conditions (DNAVision, Charleroi, Belgium). The raw ATH1 microarray data was normalized using the RMA algorithm and normalized expression values obtained (Irizarry et al., 2003a,b).

To define genes systemically up-regulated during the SAR state in Col-0 plants (SAR^+^ genes), we first calculated the ratios of the normalized expression values for the *Psm*- and mock-samples from individual SAR experiments for each gene [*P*/M]_SAR_, determined the mean values for the 3 ratios from different experiments for each gene, and selected those genes that were up-regulated by a factor of at least 3 on average. We further applied a two-sided *t*-test between the normalized expression values of the *Psm*- and those of the mock-samples for each gene and excluded those genes from our list with a *P*-value > 0.05. These two selection criteria yielded 305 genes out of the 22810 genes represented on the ATH1 GeneChip that were up-regulated by a factor of at least 3 in a statistically significant manner among the 3 individual SAR experiments (Table 1A). The number of genes up-regulated upon SAR induction on average by a factor of 5 and 10 amounted to 149 and 67, respectively. For the *fmo1* mutant, not a single SAR^+^ gene existed based on these criteria (Table 1B), corroborating our previous findings that functional *FMO1* is critical for the activation of systemic defense responses and SAR (Mishina and Zeier, 2006; Návarová et al., 2012). We also recognized that expression of several genes was consistently suppressed following SAR establishment in Col-0 plants (SAR^−^ genes), although the overall degree of gene down-regulation was lower than the degree of up-regulation (Tables 1A,B). For instance, 17 and 276 genes were significantly down-regulated by a factor of at least 4 ([*P*/M]_SAR_ < 0.25) and 2 ([*P*/M]_SAR_ < 0.5), respectively. Again, not a single SAR^−^ gene was differently expressed in 2° leaves of *Psm*- and mock-treated *fmo1* plants when following these selection criteria (Tables 1A,B).

Table 1**Number of genes up- (SAR^+^ genes) and down-regulated (SAR^−^ genes) upon *Psm*-induced SAR in Arabidopsis Col-0 and *fmo1***.

**Table:** 

**A**	**SAR**^**+**^ **(up-regulated)**		**Col-0**	***fmo1***	**B**	**SAR**^**−**^ **(down-regulated)**		**Col-0**	***fmo1***
	[*P*/M]_SAR_	>10	67	0		[*P*/M]_SAR_	<0.1	0	0
		>5	149	0			<0.25	17	0
		>3	305	0			<0.5	276	0

**Table:** 

**C**	**SAR**^**+**^ **(up-regulated)**		**Col-0**	***fmo1***	**D**	**SAR**^**−**^ **(down-regulated)**		**Col-0**	***fmo1***
	[*P*/M]_SAR_	>10	145	0		[*P*/M]_SAR_	<0.1	2	0
		>5	295	1			<0.25	50	0
		>**3**	**547**	**4**			**>0.5**	**700**	**0**

**(A,B)** [P/M]_SAR_ symbolizes the mean value over SAR experiments 1, 2 and 3 of the ratios of the normalized expression values for Psm-samples divided by those of the mock samples. A two-sided t-test comparing the normalized expression values of the Psm- and the mock-samples was performed, and genes with P > 0.05 were excluded. On the right of each table, the number of resulting **(A)** SAR^+^ genes (for [P/M]_SAR_ > 10, 5, or 3) and **(B)** SAR^−^ genes ([P/M]_SAR_ < 0.1, 0.25 or 0.5) are given. **(C,D)** Final classification of SAR^+^ genes and SAR^−^ genes. The data from the untypical SAR experiment 3 was excluded. **(C)** Only genes with mean [P/M]_SAR_ > 3 from SAR experiments 1 and 2 were considered for the SAR^+^ gene cluster. Genes whose individual [P/M]_SAR_ ratios in either experiment 1 or 2 were below 2 were excluded. These selection criteria were taken as a basis for the final classification of genes into the SAR^+^ gene cluster (number of SAR^+^ genes in bold). Number of genes with mean [P/M]_SAR_ > 5 and > 10 are also given. **(D)** Only genes with mean [P/M]_SAR_ < 0.5 from SAR experiments 1 and 2 were considered for the SAR^−^ gene cluster. Genes whose individual [P/M]_SAR_ ratios in either experiment 1 or 2 were above 0.67 were excluded (number of SAR^−^ genes in bold). Number of genes with mean [P/M]_SAR_ < 0.25 and < 0.1 are also given.

We next examined the [*P*/M]_SAR_ expression ratios of individual genes from the distinct SAR experiments more closely. Remarkably, many genes that on average belonged to the most prominently SAR-induced genes were not up-regulated in a statistically significant manner (P > 0.05) or only barely exhibited significantly increased expression values over all 3 independent experiments. Table 2 lists 10 representative genes from this group. Among them are genes such as *PR1, PR2, PR5*, and *PBS3*, which belong, according to previous results from other groups and our own findings (Sticher et al., 1997; Maldonado et al., 2002; Mishina and Zeier, 2006; Lee et al., 2007), to the most characteristic SAR^+^ genes. We recognized that many of these genes were highly induced in SAR experiments 1 and 2, but showed only a modest or low degree of up-regulation in experiment 3 (Table 2). For instance, *PR1* expression was induced by factors of 137, 183, and 7 in SAR experiments 1, 2, and 3, respectively. Thus, albeit markedly up-regulated in each of the individual SAR experiments, the high quantitative differences between expression values of experiment 3 compared to the two other experiments resulted in a P value larger than 0.05 for *PR1*. A similar trend was obvious for most of the other genes listed in Table 2. We consequently performed another two independent SAR experiments (experiments 4 and 5) and examined the expression characteristics of the 10 genes listed in Table 2 by quantitative real-time PCR analyses. Strikingly, in both newly conducted experiments, virtually all of the examined genes exhibited [*P*/M]_SAR_ ratio quantitatively similar to experiments 1 and 2. Furthermore, the [*P*/M]_SAR_ values derived from experiments 4 and 5 for the genes *PR1, AGP5, UGT76B1, LTP-like, CALM3*, and *PBS3* were quantitatively much higher than the [*P*/M]_SAR_ values obtained from experiment 3. On the basis of this data and previous findings, we concluded that the bacterial inoculation in experiment 3 only provoked a modest SAR response on the transcriptional level that is not representative for the SAR response that is generally observed.

**Table 2:** **Normalized expression values and [*P*/M]_SAR_ ratios (bold) for individual genes of the SAR experiments 1, 2 and 3, as determined by ATH1 microarray analyses**.

		**Experiment 1 (microarray)**			**Experiment 2 (microarray)**			**Experiment 3 (microarray)**			***t-test (P- value)***	**Experiment 4 (qPCR)**	**Experiment 5 (qPCR)**
		**Expression value**		**Ratio**	**Expression value**		**Ratio**	**Expression value**		**Ratio**	**[*P*1-*P*3] / [M1-M3]**	**Ratio**	**Ratio**
**Locus**	**Name**	**Mock 1**	***Psm* 1**	***[P*/M*]***_**SAR1**_	**Mock 2**	***Psm* 2**	***[P*/M*]***_**SAR2**_	**Mock 3**	***Psm* 3**	***[P*/M*]***_**SAR3**_		***[P*/M*]***_**SAR4**_	***[P*/M*]***_**SAR5**_
At2g14610	***PR1***	12.4	1703.2	**137.3**	16.1	2948.6	**183.1**	10.5	75.7	**7.2**	*0.133*	**95.9**	**194.8**
At1g02450	***NIMIN-1***	10.8	2007.9	**186.4**	13.8	1119.0	**81.3**	8.6	90.9	**10.5**	*0.128*	**75.3**	**70.2**
At4g10500	***2OGD1***	12.6	1786.1	**141.9**	14.6	1725.1	**118.0**	15.8	404.4	**25.5**	*0.046*	**78.3**	**19.5**
At1g35230	***AGP5***	9.0	956.5	**105.8**	10.4	1109.6	**106.3**	11.4	60.1	**5.3**	*0.100*	**162.3**	**26.3**
At3g11340	***UGT76B1***	7.4	798.7	**108.4**	7.6	586.1	**76.7**	6.9	14.9	**2.2**	*0.121*	**352.0**	**64.9**
At3g22600	***LTPa***	14.1	1073.8	**76.3**	14.3	1461.4	**101.9**	15.3	45.3	**3.0**	*0.116*	**78.3**	**379.5**
At3g01830	***CALM3***	7.5	544.0	**72.8**	8.7	521.2	**59.7**	7.3	51.0	**7.0**	*0.086*	**46.2**	**20.6**
At3g57260	***PR2***	48.1	1657.4	**34.5**	59.0	3412.1	**57.8**	43.2	942.1	**21.8**	*0.056*	**64.1**	**19.1**
At1g75040	***PR5***	40.3	1737.9	**43.1**	96.4	3252.2	**33.7**	29.8	367.9	**12.4**	*0.106*	**39.0**	**24.2**
At5g13320	***PBS3***	9.3	425.9	**46.1**	14.5	434.3	**29.9**	12.1	18.7	**1.5**	*0.110*	**68.6**	**35.6**

P-values resulting from a two-sided t-test between the normalized expression values of the Psm- and the mock-samples of experiments 1 to 3 are given (italic). In addition, [P/M]_SAR_ ratios of two additional, biologically independent SAR experiments that were determined by quantitative real-time PCR analyses (qPCR) are depicted.

A subsequent systematic search for differently expressed genes in the control samples of experiments 1, 2, and 3 identified about 50 genes that differed in their normalized expression values by a factor of 3 or more between experiment 3 and both other experiments. Interestingly, many of these genes represent central flavonoid pathway genes (Table 3). For instance, the normalized expression values of two main transcriptional regulators of anthocyanin biosynthesis, *MYB90* and *MYB75* (Borevitz et al., 2000), were about two and one order of magnitude, respectively, higher for the experiment 3-samples than for the samples from experiment 1 or 2. Similar quantitative expression patterns existed for *DFR* and *ANS*, encoding two key enzymes of the anthocyanidin biosynthesis pathway, dihydroflavonol reductase and anthocyanidin synthase, respectively, for the anthocyanin-5-O-glucosyltransferase gene *UGT75C1*, for the anthocyanin coumaroyltransferase gene *A3GlcCouT* and for the glutathione-S-transferase gene *GSTF12* that is involved in anthocyanin accumulation (Saito et al., 2013). Therefore, the biosynthesis of anthocyanins apparently was activated in the plants employed for experiment 3 to a markedly higher extent than in the plants from the other two experiments. Anthocyanin production in leaves is a characteristic response of plants to unfavorable environmental conditions such as drought, nitrogen deficiency or high light stress (Misyura et al., 2013). Although the plants used in experiment 3 did not exhibit a macroscopically obvious stress phenotype, it is likely that they had suffered an unexpected stress exposure prior to or in the course of the SAR experiment that resulted in the activation of anthocyanin biosynthesis. Mutual influences between pathogen defense signaling and abiotic stress pathways or the anthocyanin biosynthesis pathway do exist (Fan et al., 2009; Saijo et al., 2009), and it is possible that the moderate SAR reaction observed in experiment 3 was a consequence of such cross-talk events.

**Table 3:** **Several central anthocyanin biosynthesis genes exhibit high normalized expression values in plants of SAR experiment 3 compared with experiments 1 and 2 (M1-3: mock treatment experiment 1-3; *P*1-3: *Psm* treatment experiment 1-3)**.

**Locus**	***Name***	**Gene description**	**Expression value**						***Ratio***	
			**M1**	**M2**	**M3**	***P*1**	***P*2**	***P*3**	**M3/M1**	**M3/M2**
At1g66390	*MYB90/PAP2*	*PRODUCTION OF ANTHOCYANIN PIGMENT 2*	11.1	17.3	1281.1	14.9	12.5	539.1	*115.4*	*74.1*
At5g17220	*GSTF12*	Glutathione transferase, anthocyanin biosynthesis	14.4	19.6	721.3	15.1	54.3	284.1	*50.1*	*36.7*
At5g42800	*DFR*	Dihydroflavonol reductase	12.0	12.7	497.6	12.1	21.7	117.9	*41.4*	*39.2*
At4g22880	*ANS*	Anthocyanidin synthase, leucoanthocyanidin dioxygenase	17.5	31.4	687.7	17.8	46.1	134.4	*39.3*	*21.9*
At4g32940	*γ-VPE*	Vacuolar cysteine proteinase	220.0	315.7	3362.8	247.3	315.2	2297.1	*15.3*	*10.7*
At5g54060	*UF3GT*	*UDP-GLC-FLAVONOID 3-O-GLUCOSYLTRANSFERASE*	15.2	16.4	192.1	19.3	18.3	47.8	*12.7*	*11.7*
At1g03495	*A3GlcCouT*	Anthocyanin coumaroyltransferase	9.9	15.4	142.3	10.4	15.2	45.6	*14.3*	*9.2*
At4g14090	*UGT75C1*	Anthocyanidin-5-O-glucosyltransferase	10.6	9.1	86.7	10.5	9.2	28.2	*8.1*	*9.5*
At1g56650	*MYB75/PAP1*	*PRODUCTION OF ANTHOCYANIN PIGMENT 1*	109.7	117.4	803.9	28.7	26.1	481.8	*7.3*	*6.8*
At1g62710	*β-VPE*	Vacuolar cysteine proteinase	25.5	36.4	188.4	17.0	32.8	104.5	*7.4*	*5.2*
At3g51240	*F3'H*	Flavanone 3-hydroxylase	155.6	338.4	1473.2	128.6	271.5	544.6	*9.5*	*4.4*
At5g13930	*CHS*	Chalcone synthase (*TRANSPARENT TESTA 4, TT4*)	26.8	410.9	1146.6	93.6	280.5	334.9	*42.7*	*2.8*
At1g65060	*4CL3*	4-coumarate-CoA ligase	15.1	31.9	102.7	22.0	27.3	22.0	*6.8*	*3.2*
At5g05270	*CHI*	Chalcone isomerase	10.8	39.3	84.3	20.0	23.4	23.1	*7.8*	*2.1*

Since the transcriptional data obtained from SAR experiment 3 markedly differed from those resulting from the other four experiments, we decided to exclude this data set from further analyses. We defined new selection criteria to classify the SAR^+^ genes and only considered genes whose mean [*P*/M]_SAR_ ratios from experiments 1 and 2 were larger than 3, and whose individual [*P*/M]_SAR_ ratios in either experiment was at least 2. We thus ensured that a marked up-regulation of the selected SAR^+^ genes had taken place in each of the experiments 1 and 2. When following this procedure, all the characteristically SAR up-regulated genes listed in Table 2 fell into the category “SAR^+^ genes” which altogether consisted of 547 genes for the Col-0 wild-type (Table 1C). Thereof, 295 and 145 SAR^+^ genes showed average [*P*/M]_SAR_ ratios larger than 5 and 10, respectively (Table 1C). To classify genes down-regulated during SAR, we selected genes with average [*P*/M]_SAR_ ratios lower than 0.5, whereby genes with rations higher than 0.67 in either experiment 1 or 2 were excluded. This procedure yielded a group of 700 SAR^−^ genes, from which 50 genes had average [*P*/M]_SAR_ ratios lower than 0.25 and 2 genes exhibited average [*P*/M]_SAR_ ratios lower than 0.1 (Table 1D). The new selection criteria pinpointed only 4 genes systemically up-regulated in *fmo1* by a factor of at least 3 after *Psm*-infection. Moreover, not a single down-regulated gene was assigned for *fmo1* (Tables 1C,D), again highlighting that *fmo1* is virtually non-responsive to pathogen stimuli at the systemic plant level.

**Figure 1:**
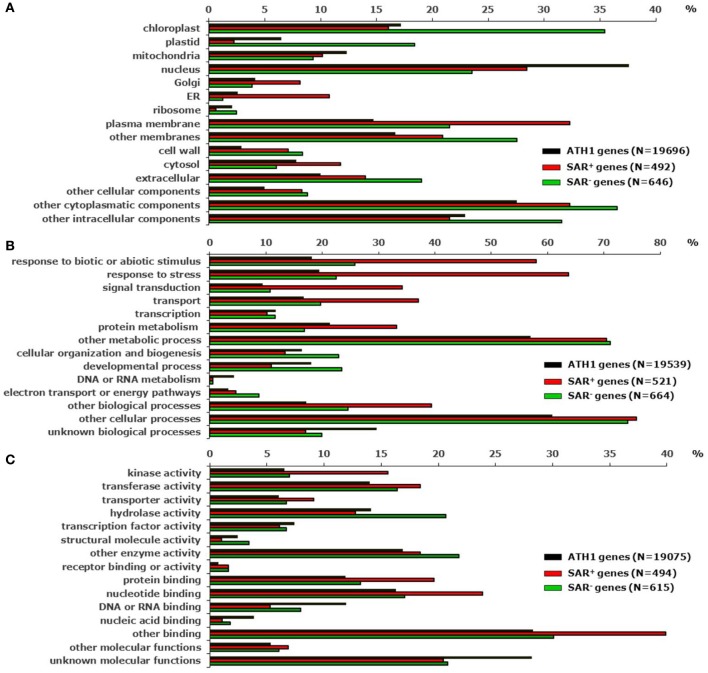
**Functional categorization of SAR^+^ and SAR^−^ genes according to Gene Ontology (GO) descriptions (http://www.arabidopsis.org/tools/bulk/go/index.jsp)**. The 547 genes up-regulated during SAR by [*P*/M]_SAR_ > 3 (SAR^+^ genes) (Table 1C), the 700 genes down-regulated during SAR by [*P*/M]_SAR_ < 0.5 (SAR^−^ genes) (Table 1D), and all the genes (22810) represented on the ATH1 chip were set as input lists for the categorization algorithm. The depicted value on each of the x axes represents the quotient of “the number of genes annotated to terms of the respective categorization class” divided by “the total number of genes from the input list annotated to any term in this ontology (N)” in %. **(A)** Categorization class “cellular component”. **(B)** Categorization class “biological process”. **(C)** Categorization class “molecular function”.

As a first step to functionally characterize the two clusters of SAR^+^ and SAR^−^ genes, we used the The Arabidopsis Information Resource (TAIR) functional categorization tool to classify the genes according to Gene Ontology (GO) descriptions which discriminates the three main classes “cellular component”, “biological process” and “molecular function” (http://www.arabidopsis.org/tools/bulk/go/index.jsp). We thereby compared the SAR^+^ (547) and the SAR^−^ (700) gene cluster with all the genes represented on the ATH1 Genechip (22810) (Tables 1C,D). In the class “cellular component”, the functional categories “Golgi”, “endoplasmatic reticulum (ER)”, and “plasma membrane” were strongly overrepresented in the group of SAR^+^ genes. Moreover, the categories “cell wall”, “extracellular”, and “cytosol” were moderately overrepresented among the SAR^+^ genes, whereas the categories “plastid”, “nucleus”, and “ribosome” were underrepresented (Figure 1A). For the group of SAR^−^ genes, it became apparent that “chloroplast”, “plastid”, “cell wall”, and “extracellular” were highly represented categories (Figure 1A). These tendencies might indicate that cellular processes associated with the secretory apparatus and extracellular defenses are activated during SAR, and that certain activities occurring in chloroplasts and plastids are reduced. Not unexpectedly, in the class “biological process”, genes with GO annotations “response to (a)biotic stimuli” and “response to stress” were typically found in the group of SAR up-regulated genes (Figure 1B). Furthermore, the functional categories “signal transduction”, “transport”, and “protein metabolism” were highly represented in the group of SAR^+^ genes, whereas the categories “developmental processes” and “DNA or RNA metabolism” were underrepresented. The category “electron transport and energy pathways” was relatively prominent among the SAR^−^ genes (Figure 1B). Thus, the relative distributions of GO categories from the class “biological processes” indicated that stimulus- and stress-related signal transduction events, transport processes and protein metabolism are prominent features of the SAR state, whereas certain developmental, nucleic acid metabolic, and energy-related events might be reduced in SAR-induced plants. From the GO categories grouped into the class “molecular function”, “kinase activity”, “protein binding”, “nucleotide binding”, “receptor binding”, and “transporter activity” were overrepresented in the SAR^+^ gene cluster, whereas “DNA or RNA/nucleic acid binding” was poorly represented (Figure 1C). Again, this might emphasize the importance of signal transduction events such as protein phosphorylation and protein-ligand interactions for SAR.

In the cluster of SAR^−^ genes, the relatively high abundance of the GO annotation “hydrolase activity” was one of the most obvious features (Figure 1C). Moreover, when examining the specific functional annotation of genes in this cluster, two other trends became apparent. First, several genes belonging to the xyloglucan endotransglucosylase/hydrolase (XTH), the (fasciclin-like) arabinogalactan protein (AGP), the expansin-like protein, the extensin-like protein, and the polygalacturonase families were among the genes most strongly down-regulated during SAR (Table 4). Members of these gene families encode proteins associated with the extracellular matrix and/or the cell wall and have important functions in the rearrangement of cell wall components, wall loosening, cell elongation, and cell growth. For instance, members of the XTH family are involved in xyloglucan endotransglucosylation and/or in xyloglucan hydrolysis. These enzymatic activities can contribute to primary cell growth by restructuring and loosening the xyloglucan network, thereby enabling cell expansion (Rose et al., 2002). Two of the four XTH genes markedly down-regulated during SAR, *XTH4* and *XTH31* (Table 4), code for proteins that have been experimentally identified as constituents of the cell wall proteome in Arabidopsis hypocotyls (Irshad et al., 2008). The strongly SAR down-regulated *EXLA1* gene belongs to the expansin multigene family. The presence of EXLA1 protein in the Arabidopsis cell wall has also been experimentally verified (Irshad et al., 2008). Expansins directly modify the mechanical properties of plant cell walls leading to turgor-driven cell extension (Li et al., 2002). Another class of extracellular proteins implicated in plant growth and development are the hydroxyproline-rich and highly glycosylated AGPs (Schultz et al., 2002). Several AGPs, among them the three fasciclin-like AGPs (FLAs) *FLA8, FLA9*, and *FLA13*, belong to the genes most highly down-regulated following SAR induction (Table 4). FLAs have, in addition to predicted AGP-like glycosylated regions, putative cell adhesion domains known as fasciclin domains (Johnson et al., 2003). Together, these examples indicate that the SAR state is associated with a marked down-regulation of various genes involved in cell wall modification, cell growth and development (Table 4).

**Table 4:** **Genes associated with cell wall remodelling, cell extension, and growth belong to the most strongly down-regulated genes during SAR**.

**Locus**	***Name***	**Gene description**	**[*P/*M]**_**SAR**_	**Rank among down-regulated genes**
At3g45970	*EXLA1*	Expansin-like A1^*^	0.14	11
At1g03870	*FLA9*	Fasciclin-like arabinogalactan-protein 9	0.17	13
At2g45470	*FLA8*	Fasciclin-like arabinogalactan-protein 8	0.18	15
At2g06850	*XTH4*	Xyloglucan endotransglucosylase/hydrolase^*^	0.18	16
At3g06770	−	polygalacturonase (pectinase)^*^	0.20	17
At1g55330	*AGP21*	Arabinogalactan protein 21	0.21	22
At2g14890	*AGP9*	Arabinogalactan protein 9 /putative proline-rich protein	0.21	23
At2g47930	*AGP26*	Arabinogalactan protein 26	0.22	26
At1g12090	*ELP*	Extensin-like protein	0.23	32
At5g44130	*FLA13*	Fasciclin-like arabinogalactan-protein 13	0.24	39
At3g44990	*XTH31*	Xyloglucan endotransglucosylase/hydrolase^*^	0.24	43
At4g37800	*XTH7*	Xyloglucan endotransglucosylase/hydrolase	0.25	52

Genes with asterisks encode proteins experimentally verified as cell wall constituents.

The cluster of SAR^−^ genes also contained several genes involved in glucosinolate and sinapoylester production (Table 5). These include genes encoding regulatory components or enzymes of indolic glucosinolate biosynthesis such as the MYB transcription factor MYB34 and the cytochrome P450 CYP79B3 that converts Trp to the indolic glucosinolate precursor indole-3-acetaldoxime (Glawischnig et al., 2004; Celenza et al., 2005), and of aliphatic glucosinolate biosynthesis such as the flavin-dependent monooxygenases FMO_GS−OX1_ and FMO_GS−OX3_ that oxidize Met-derived methylthioalkyl glucosinolates to methylsulfinylalkyl glucosinolates (Li et al., 2008), or the 2-oxoglutarate-dependent dioxygenase AOP2 involved in the conversion of methylsulfinylalkyl to alkenyl glucosinolates (Table 5A) (Neal et al., 2010). Moreover, among the most prominently SAR down-regulated genes are *SCPL8, SCPL10*, and *SCPL13* (Table 5B), encoding serine carboxypeptidase-like proteins that act as sinapoyltransferases to generate sinapoylmalate, sinapoylanthocyanins, and 1,2-disinapoyl-glucose derivatives, respectively (Stehle et al., 2009; Fraser and Chapple, 2011). Sinapoylesters and glucosinolates are among the most abundant secondary metabolites produced in Arabidopsis in the course of normal growth and development (Stehle et al., 2009; Sønderby et al., 2010). The reduced expression of genes involved in the constitutive production of major secondary metabolites in SAR-induced compared with control plants again supports the hypothesis that SAR represents a state of diminished vegetative growth.

**Table 5:** **Genes involved in the biosynthesis of major constitutively produced secondary metabolites are down-regulated during SAR**.

	**Locus**	***Name***	**Gene description**	***P/*M**	**Rank among down-regulated genes**
**A**					
	At5g60890	*MYB34/ATR1*	MYB transcription factor, ALTERED TRYPTOPHAN REGULATION 1, regulates indole glucosinolate biosynthesis	0.25	51
	At4g03060	*AOP2*	2-oxoglutarate-dependent dioxygenase, ALKENYL HYDROXALKYL PRODUCING 2, aliphatic glucosinolate biosynthesis, conversion of methylsulfinylalkyl glucosinolates to alkenyl glucosinolates, not functional in Col-0	0.27	72
	At1g65860	*FMO GS-OX1*	FLAVIN-MONOOXYGENASE GLUCOSINOLATE S-OXYGENASE 1, aliphatic glucosinolate biosynthesis, conversion of methylthioalkyl glucosinolates to methylsulfinylalkyl glucosinolates	0.34	163
	At1g62560	*FMO GS-OX3*	FLAVIN-MONOOXYGENASE GLUCOSINOLATE S-OXYGENASE 3, aliphatic glucosinolate biosynthesis, conversion of methylthioalkyl glucosinolates to methylsulfinylalkyl glucosinolates	0.36	215
	At4g03070	*AOP1*	2-oxoglutarate-dependent dioxygenase, similar to AOP2, possibly involved in aliphatic glucosinolate biosynthesis	0.40	339
	At2g22330	*CYP79B3*	Cytochrome P450 monooxygenase, converts Trp to indole-3-acetaldoxime (IAOx), a precursor to IAA and indole glucosinolates	0.41	370
**B**					
	At2g22980	*SCPL13*	Serine carboxypeptidase-like (SCPL) protein; sinapoylglucose:sinapoylglucose sinapoyltransferase	0.11	5
	At2g23000	*SCPL10*	SCPL protein; anthocyanin sinapoyltransferase	0.22	28
	At2g22990	*SCPL8/SNG1*	SCPL protein; sinapoylglucose:malate sinapoyltransferase	0.30	100

**(A)** Genes associated with glucosinolate biosynthesis. **(B)** Sinapoyltransferase genes involved in sinapoylester biosynthesis.

Together, the marked down-regulation of genes with presumed roles in cell wall modification, cell growth and the constitutive production of secondary metabolites (Tables 4, 5), the overrepresentation of annotated chloroplast functions among SAR down-regulated genes (Figure 1A), and the strong up-regulation of stimulus-, stress- and defense-related genes during SAR (Figure 1B) indicate that, compared with control plants, SAR-induced plants reallocate a part of their physiological activity from vegetative growth towards particular defense-associated processes that confer broad-spectrum disease resistance (see below).

### Regulatory principles underlying SAR gene expression

Having categorized defined groups of SAR up- and down-regulated genes, we next aimed to combine our SAR expression data with further transcriptional information to elucidate regulatory principles that govern the SAR response. We used the FIRe software, an Excel® macro designed for rapid microarray data analysis (Garcion et al., 2006), to assemble the SAR expression data with expression data from other, publicly available microarray experiments describing the impact of various defense-, stress-, and hormone-related stimuli on gene expression in Arabidopsis plants. Information about the employed microarray data, the experimenters, and the experimental setup underlying each data set are summarized in Table 6.

**Table 6:** **Publicly available microarray data sets (“microarrays 1–7”) used in this study**.

**Microarray**	**Name**	**Experimenter**	**Experiment description**	**Designation**	**Depicted value**
0	NASCARRAYS-703	Griebel, Attaran, Zeier	Biological SAR, syringe infiltration of lower leaves of 5 week-old Col-0 plants with *Psm* (OD 0.005), upper (non-treated) leaves harvested 2 d later, plants grown in soil under a 9/15-h light/dark cycle at 21/18 °C	“SAR“	*Psm*/mock = [P/M]_SAR_
1	NASCARRAYS-392	Wang, Dong (Wang et al., 2006)	60 μM BTH, spray-treatment of 4 week-old Col-0 plants grown on soil under a 16/8-h light/dark cycle at 22°C, samples 24 h post treatment were considered	“BTH”	BTH/mock = [S/M]_BTH_
2a	NASCARRAYS-454	Mitra, Glazebrook (Wang et al., 2008)	Leaf inoculation (syringe infiltration) of 4-5 week-old Col-0 plants with *Psm* (OD 0.002), inoculated leaves harvested 24 hpi, plants grown in soil under a 12/12-h light/dark cycle at 22 °C	“*Psm*”	Col-0-*Psm*/Col-0-mock = [S/M]_Psm_
2b	NASCARRAYS-454	Mitra, Glazebrook (Wang et al., 2008)	Leaf inoculation (syringe infiltration) of 4-5 week-old Col-0 or mutant plants with *Psm* (OD 0.002), inoculated leaves harvested 24 hpi, plants grown in soil under a 12/12-h light/dark cycle at 22 °C	“Col */ mutant*”	Col-0-*Psm*/*mutant*-*Psm* = [Col/*mutant*]_Psm_
3	E-GEOD-3984	Thibaud-Nissen (Thibaud-Nissen et al., 2006)	1 mM SA in 0.01 % Silwet, spray-treatment of 3-4 week-old, non-flowering Col-0 plants, leaf samples harvested 2 h post treatment	“SA”	SA/mock = [S/M]_SA_
4	NASCARRAYS-174	Goda, Yoshida, Shimada (Goda et al., 2008)	7 day-old Col-0 seedlings grown in MS liquid medium under constant light at 22°C were treated with 10 μM MeJA, leaf samples at 3 h post treatment were considered	“JA”	JA/mock = [S/M]_JA_
5	NASCARRAYS-338	Mittler (Davletova et al., 2005)	Application of 20 mM H_2_O_2_ to 5 day-old Col-0 seedlings grown on MS agar plates under constant light at 21-22° C	“H_2_O_2_”	H_2_O_2_/mock = [S/M]_H2O2_
6	NASCARRAYS-176	Goda, Yoshida, Shimada (Goda et al., 2008)	7 day-old Col-0 seedlings grown in MS liquid medium under constant light at 22° C were treated with 10 μM ABA, leaf samples at 3 h post treatment were considered	“ABA”	ABA/mock = [S/M]_ABA_
7	NASCARRAYS-123	Scheel, Brunner, Westphal	Surface-treatment of leaves of 5 week-old Col-0 plants with 1 mM flg22 peptide, plants grown on soil at 22°C under a 8/16 hour light/dark regime, leaf samples 4 h post treatment were considered	“flg22”	flg22/mock = [S/M]_flg22_

Sources, experimenters, relevant literature citations, and experimental descriptions are given.

**Figure 2:**
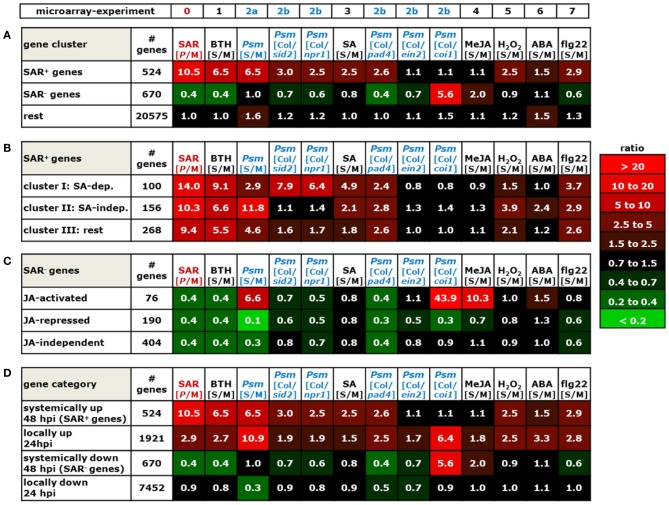
**Mean values of [*P*/M]_SAR_, [S/M]_stimulus_, and [Col/*mutant*]_Psm_ ratios over groups of differently categorized genes**. The numerical values are embedded in a heat map, and the legend on the right hand side depicts the value range assigned to each color of the heat map. The top row indicates the origin of the microarray data (Table 6). The selection criteria for the categorization of genes are detailed in the main text. **(A)** SAR^+^ genes, SAR^−^ genes, and remaining ATH1 genes. Gene probes not unequivocally assignable to a single gene (“s_at”-probes) were removed so that the number of genes was slightly reduced in each group (Tables 1C,D). (B) SA-dependent (cluster I), SA-independent (cluster II), and remaining (cluster III) SAR^+^ genes. **(C)** JA-activated, JA-repressed, and JA-independent SAR^−^ genes. **(D)** Comparison of expression characteristics of SAR^+^ genes (row 1) and SAR^−^ genes (row 3) with locally up-regulated genes ([S/M]_Psm_ > 3, row 2) and locally down-regulated genes ([S/M]_Psm_ < 0.5, row 4).

**Figure 3:**
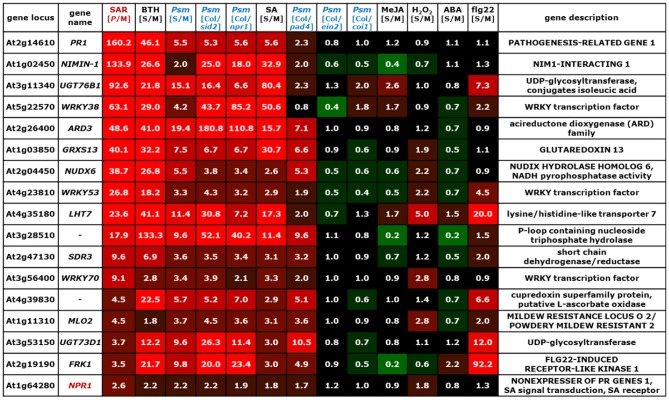
**Values of [*P*/M]_SAR_, [S/M]_stimulus_, and [Col/*mutant*]_Psm_ ratios for individual genes of the group of SA-dependent (cluster I) SAR^+^ genes (Figure 2B)**. The data for the *NPR1* gene is also included into the list. The gene names of genes indispensable for SAR are depicted in red. The legend for the heat map representation is depicted in Figure 2.

**Figure 4:**
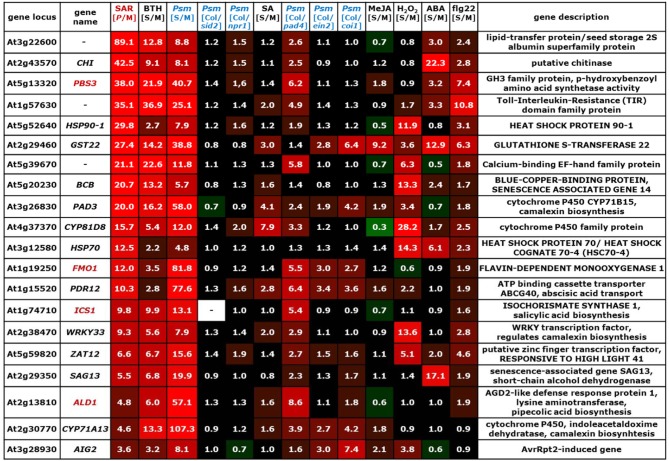
**Values of [*P*/M]_SAR_, [S/M]_stimulus_, and [Col/*mutant*]_Psm_ ratios for individual genes of the group of SA-independent (cluster II) SAR^+^ genes (Figure 2B)**. The gene names of genes indispensable for SAR are depicted in red. The legend for the heat map representation is depicted in Figure 2.

**Figure 5:**
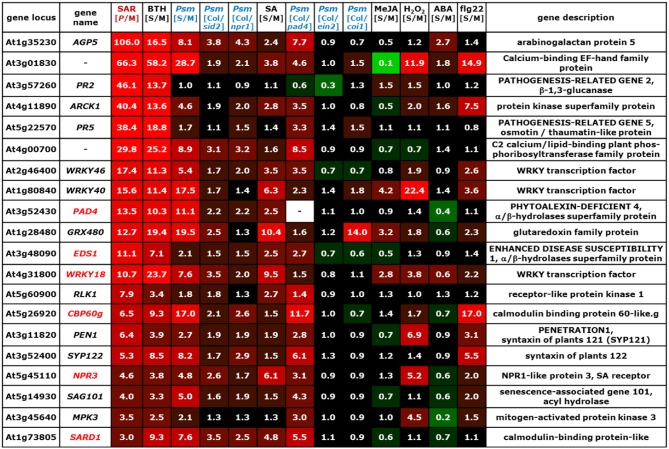
**Values of [*P*/M]_SAR_, [S/M]_stimulus_, and [Col/*mutant*]_Psm_ ratios for individual genes of the group of partially SA-dependent (cluster III) SAR^+^ genes (Figure 2B)**. The gene names of genes indispensable for SAR are depicted in red. The legend for the heat map representation is depicted in Figure 2.

**Figure 6:**
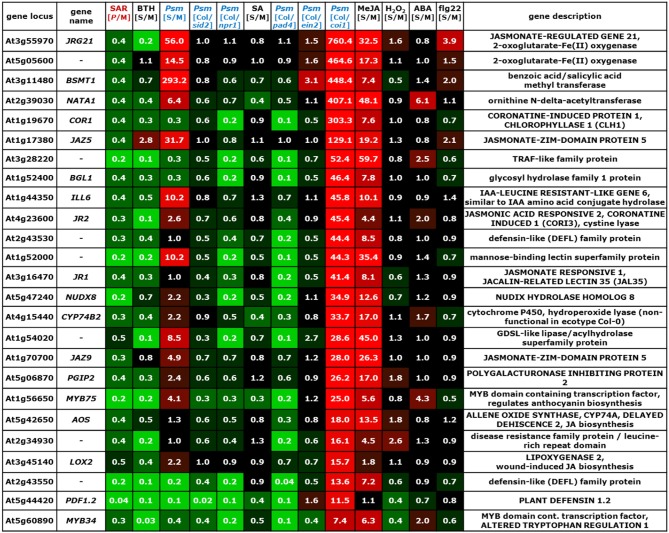
**Values of [*P*/M]_SAR_, [S/M]_stimulus_, and [Col/*mutant*]_Psm_ ratios for individual genes of the group of JA-activated SAR^−^ genes (Figure 2C)**. The legend for the heat map representation is depicted in Figure 2.

**Figure 7:**
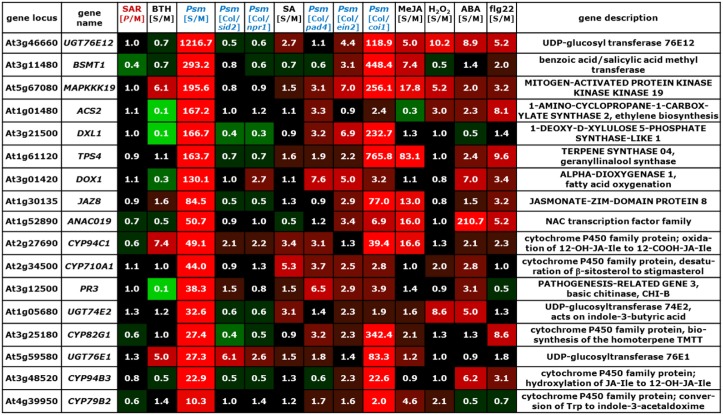
**Values of [*P*/M]_SAR_, [S/M]_stimulus_, and [Col/*mutant*]_Psm_ ratios for individual genes strongly up-regulated at inoculation sites (24 hpi) but not in distal tissue (48 hpi) (Figure 2D)**. The legend for the heat map representation is depicted in Figure 2.

The designation of each experiment and the value depicted in the Figures 2–7 are also indicated.

The information drawn from these microarray experiments is based on two distinct types of comparisons. In most experiments, Arabidopsis wild-type plants were exogenously treated with a chemical stimulus and the gene expression values of stimulus-treated plants or leaf tissue was compared to the values resulting from an adequate mock-treatment. In this way, we could acquire information about the impact of exogenous application of defense and stress hormones or their derivatives [SA, methyl jasmonate (MeJA), abscisic acid (ABA)], oxidative stress (H_2_O_2_), the resistance-enhancing chemical BTH, which is often considered as an SA analogue (Lawton et al., 1996; Wang et al., 2006; Canet et al., 2010), and flg22-treatment, a 22mer peptide corresponding to the elicitor-active domain of the bacterial PAMP flagellin (Gomez-Gomez et al., 1999), on gene expression (Table 6, Figures 2–7; microarray designations “BTH”, “SA”, “MeJA”, “H_2_O_2_”, “ABA”, “flg22”). Similarly, one experiment investigated the impact of *Psm* inoculation on gene expression (designation “*Psm*”). In contrast to our SAR microarray data (designation “SAR”) that describes systemic changes in 2°, non-inoculated leaves at 48 hours post inoculation (hpi), this experiment yielded information about the local changes in gene expression at the site of pathogen inoculation (1° leaves) at 24 hpi. For all microarray experiments, we calculated the means of normalized expression values from the stimulus replicates and divided them by the means of the respective mock-values. This yielded, in analogy to the [*P*/M]_SAR_ ratios for the SAR experiment, stimulus to mock ratios [S/M]_stimulus_ that quantitatively indicated by which factors genes were differently expressed following application of the exogenous stimulus compared with the mock-control in wild-type Col-0 plants (Figures 2–7).

The microarray experiment 2 (Table 6) that investigated the impact of *Psm* leaf inoculation on local gene expression yielded two kinds of information: the ratio *Psm*/mock in Col-0 ([S/M]_Psm_; microarray 2a), and expression ratios of *Psm*-treated wild-type samples to different *Psm*-treated mutant samples ([Col/*mutant*]_Psm_; microarray 2b). We evaluated the expression data from *sid2* which is defective in *ICS1* and, consequently, pathogen-induced SA production (Nawrath and Métraux, 1999; Wildermuth et al., 2001), SA insensitive *npr1* defective in the transcriptional co-activator and SA receptor NPR1 (Durrant and Dong, 2004; Wu et al., 2012), *pad4* defective in the lipase-like defense regulator PHYTOALEXIN-DEFICIENT4 (Jirage et al., 1999), ethylene-insensitive *ein2* (Alonso et al., 1999), and JA insensitive *coi1* defective in the JA receptor CORONATINE INSENSITIVE1 (Katsir et al., 2008). The [Col/*mutant*]_Psm_ ratios could be used to assess at the genetic level whether *Psm*-induced gene expression was dependent on SA accumulation (*sid2*), SA perception (*npr1*), JA perception (*coi1*), and ET perception (*ein2*) (Figures 2–7).

We now assembled the [*P*/M]_SAR_ ratios for each gene from the SAR experiment with the corresponding [S/M] or [Col/*mutant*]_Psm_ ratios from the other microarray experiments using the “merge” macro of the FIRe program (Garcion et al., 2006). This yielded an Excel® table in which the gene expression information from all the experiments listed in Table 6 for the ATH1 genes was brought together. Some Affymetrix probes (labeled “s_at”) hybridize to two or more related genes (Redman et al., 2004). These non-gene specific gene probes had been eliminated in some of the public microarray data used, and we consequently also deleted them from our merged Excel® list. The genes from our list were then grouped into different categories according to evaluation criteria outlined below (Figure 2), and excerpts of the Excel® data set relating to these categories are depicted in Figures 3–7. In addition to [*P*/M]_SAR_ ratios, the normalized expression values of the genes depicted in Figures 3–7 for each of the three replicate SAR experiments is provided in an accompanying Excel® data file (Supplemental material).

Before considering above-mentioned gene categories, however, we discuss expression information of a specific example, the classical SAR marker gene *PR1* (Figure 3, top row), to illustrate the gene regulatory information that we have drawn from the merged data set. *PR1* is the most prominently up-regulated SAR^+^ gene ([*P*/M]_SAR_ = 160.2), and its expression is, as reported in previous studies (Delaney et al., 1995; Lawton et al., 1996), enhanced by exogenous BTH ([S/M]_BTH_ = 46.1) and SA ([S/M]_SA_ = 5.6) (Figure 3). Local inoculation with *Psm* also increases *PR1* expression at 24 hpi, leading to an ([S/M]_Psm_ ratio of 5.5 in the “*Psm*” experiment. The [Col/*sid2*]_Psm_ (5.3) and the [Col/*npr1*]_Psm_ (5.6) ratios reveal that *PR1* expression upon *Psm*-inoculation in the two mutants does not exceed *PR1* expression in Col-0 mock-control plants, indicating that *P. syringae*-induced *PR1* expression fully depends on ICS1-mediated SA biosynthesis and on NPR1-mediated downstream signaling. Thus, exogenous SA is sufficient and endogenous SA accumulation following bacterial inoculation is necessary to induce *PR1* expression. By contrast, induction of *PR1* expression is independent of JA signaling, because exogenous MeJA ([S/M]_JA_ = 1.2) does not elevate *PR1* levels, and the [Col/*coi1*]_Psm_ ratio equals 1.0, indicating identical *P. syringae*-induced expression of the gene in the Col-0 wild-type and in JA-insensitive *coi1*. Further, the [Col/*pad4*]_Psm_ ratio equals 2.3, indicating an attenuated but not a fully compromised *Psm*-induced expression of *PR1* in *pad4* and thus a partial PAD4 dependency (Figure 3). Finally, *PR1* expression occurs virtually independently of ET signaling ([Col/*ein2*]_Psm_ = 0.8) and is not stimulated by exogenous H_2_O_2_ ([S/M]_H2O2_ = 0.9), ABA ([S/M]_ABA_ = 1.1), or flg22 ([S/M]_flg22_ = 1.1) (Figure 3).

To draw information about regulatory principles of gene expression in the clusters of SAR up-regulated, SAR down-regulated, and remaining ATH1 genes, we first determined the mean values of [*P*/M]_SAR_, [S/M]_stimulus_, and [Col/*mutant*]_Psm_ ratios for all the genes from each category. Compared with the rest of the ATH1 genes, the SAR^+^ genes exhibited, in addition to a strong average expression in 2° leaves of SAR-induced plants (mean [*P*/M]_SAR_ = 10.5), a marked average up-regulation in leaves of BTH-treated plants and in *Psm*-inoculated leaves (Figure 2A). To a lesser extent, the average expression of these genes was stimulated by the SA pathway, H_2_O_2_, and flg22, and positively influenced by functional *PAD4*. Moreover, a small average inducing stimulus of ABA on the expression of SAR^+^ genes was obvious, and the JA- and ET- pathways had virtually no influence on the average SAR^+^ gene expression patterns. By contrast, the genes down-regulated in SAR (mean [*P*/M]_SAR_ = 0.4) exhibited a completely different regulatory pattern. On average, these genes were markedly down-regulated by BTH and PAD4, and to lesser extent, by SA signaling, ET-signaling, and flg22-treatment. Remarkably, the average expression of the SAR^−^ genes was strongly stimulated by JA signaling ([Col/*coi1*]_Psm_ = 5.6) (Figure 2A).

The positive effect of BTH, SA signaling, and PAD4 on the average expression of SAR^+^ genes is consistent with the facts that BTH induces plant resistance patterns similar to SAR (Lawton et al., 1996), that SA is a central signal for SAR (Wildermuth et al., 2001; Mishina and Zeier, 2006), and that the PAD4 defense regulator is required for SAR establishment (Mishina and Zeier, 2006; Jing et al., 2011). One of the hallmarks of SAR is systemic SA accumulation at the onset of SAR (Métraux et al., 1990; Shulaev et al., 1995; Attaran et al., 2009), and increased levels of SA in 2° leaves upon SAR induction is closely associated with increased expression of SAR-related genes (Shulaev et al., 1995; Mishina and Zeier, 2006). We therefore determined whether all or only a sub-fraction of the SAR^+^ genes are indeed up-regulated by SA. To categorize SA-regulated genes, we aimed at selecting only those genes whose induced local expression upon *Psm*-treatment was severely compromised in *sid2*. We therefore had to consider genes with increased [Col/*sid2*]_Psm_ ratios and first selected genes with [Col/*sid2*]_Psm_ ratios > 2. However, this criterion alone was not sufficient for selection because the genes strongly varied in their [S/M]_Psm_-ratios and thus their *Psm*-responsiveness. For instance, genes strongly *Psm*-up-regulated in Col-0 still exhibit considerable *Psm*-induced up-regulation in *sid2* with the criterion [Col/*sid2*]_Psm_ ratios > 2 and thus are only weakly SA dependent. We consequently coupled the [Col/*sid2*]_Psm_ ratio to the degree of *Psm*-responsiveness and defined that only genes with quotients of [Col/*sid2*]_Psm_ / [S/M]_Psm_ > 0.67 were taken. The combination of these two selection criteria provided a set of 100 genes out of 541 SAR^+^ genes whose expression was locally *Psm*-inducible in a modest to strong manner and whose *Psm*-induced up-regulation was largely dependent on *SID2/ICS1* and thus on endogenous SA. Moreover, most of these genes were also up-regulated by exogenous SA (Figures 2B, 3). To categorize SAR^+^ genes independently expressed from SA, we assembled all the genes with a low [Col/*sid2*]_Psm_ ratio ([Col/*sid2*]_Psm_ < 1.5) that were up-regulated upon *Psm* inoculation by at least a factor of 3. This selection yielded 156 SA-independent SAR^+^ genes (Figures 2B, 4). The remaining SAR^+^ genes (268) were grouped into a third category that mainly consisted of genes partly requiring *SID2/ICS1* for *Psm*-induced expression (partly SA-dependent genes), or of genes not locally up-regulated by *Psm* at 24 hpi (Figures 2B, 5). Therefore, SAR^+^ genes were categorized into three groups according to their SA-dependent expression: strictly SA-dependent genes (cluster I), SA-independent genes (cluster II), and cluster III genes predominantly consisting of genes with partial SA-dependency. Irrespective of their SA responsiveness, the vast majority of SAR^+^ genes were also up-regulated by exogenous BTH, indicating that the action of the so-called “SA analogue” BTH on gene transcription is significantly broader than the action of SA itself (Figures 2B, 3, 4, 5). Similarly, genes down-regulated during biological SAR were generally down-regulated by exogenous BTH (Figures 2, 6). These tendencies indicate extensive overlap between the biologically-induced SAR state and the state of enhanced disease resistance after BTH application. Nevertheless, some differences between biological SAR and BTH-treatment existed for the transcription levels of individual genes not affected in biological SAR (Figure 2A, “rest”). Hereunder, 1.8 % of genes were positively ([S/M]_BTH_ > 3) and 15.8 % negatively ([S/M]_BTH_ < 2) regulated by BTH (specific examples are *MAPKKK19, CYP94C1, UGT76E1, ACS2, DXL1, PR3*; Figure 7).

On average, the [Col/*sid2*]_Psm_ ratios for the SA-dependent SAR^+^ genes, the SA-independent SAR^+^ genes, and the remaining genes amounted to 7.9, 1.1, and 1.6, respectively, reflecting strong, virtually absent, and moderate SA regulation of the respective genes (Figure 2B). The [Col/*npr1*]_Psm_ ratios (6.4, 1.4, 1.7) paralleled the [Col/*sid2*]_Psm_ ratios, confirming above-mentioned influences of the SA pathway on the regulation of the different gene groups (Figure 2B). The [S/M]_SA_ values exhibited a similar tendency as well (4.9 for SA-dependent, 2.1 for SA-independent, and 1.8. for remaining genes), although some genes of the SA-independent gene cluster showed a moderate responsiveness to exogenous SA (Figure 2B). *PAD3* is a typical example of a gene grouped into the SA-independent gene cluster which responded to exogenous SA (Figure 4). We reasoned, however, that in such cases, a [Col/*sid2*]_Psm_ ratio close to 1 would provide a more meaningful criterion for SA-independency than an elevated [S/M]_SA_ value, because the [Col/*sid2*]_Psm_ ratio results from physiological differences in SA rather than from artificial SA differences caused by exogenous treatment. In general, however, the [Col/*sid2*]_Psm_ values paralleled the [S/M]_SA_ values remarkably well: SA-dependent genes generally exhibited high values for both parameters (Figure 3), and the majority of SA-independent genes, as exemplified by *CHI, FMO1*, and *SAG13*, showed both [Col/*sid2*]_Psm_ and [S/M]_SA_ values close to 1 (Figure 4).

Noticeably, the average [*P*/M]_SAR_ ratios and the [S/M]_flg22_ ratios were higher for the SA-dependent than for the SA-independent SAR^+^ genes, indicating a comparable high degree of up-regulation of SA-regulated genes upon both SAR induction and flg22-treatment (Figure 2B). When examining the expression patterns of individual SA-dependent SAR^+^ genes, it became apparent that several genes such as *FRK, LHT7*, or *UGT73D1* are strongly flg22-responsive whereas others are not at all (Figure 3). In addition, the average induction of SA-independent SAR^+^ genes was higher than the induction of SA-dependent SAR^+^ genes in *Psm*-inoculated tissue, in H_2_O_2_-treated tissue, and in ABA-treated tissue (Figure 2B), indicating a more prominent stimulatory capacity of H_2_O_2_- and ABA-signaling on the expression of individual members of the SA-independent compared with the SA-dependent gene cluster (Figures 2B, 3, 4).

### SAR^+^ genes exhibiting tight SA regulation (SAR^+^ gene cluster I)

Representative examples of SAR^+^ genes tightly regulated by SA are *PR1*, the classical marker gene for SA-dependent defense gene activation (Nawrath and Métraux, 1999), *NIMIN-1, UGT76B1, WRKY38, GRXS13, NUDX6, SDR3, WRKY70*, and *MLO2* (Figure 3). *NPR1*, encoding a critical regulator of SAR that functions in SA perception and transcriptional activation of downstream genes, is only moderately up-regulated in 1° and 2° leaf tissue upon inoculation ([*P*/M]_SAR_ = 2.6) and thus not assigned to the group of SAR^+^ genes. Nevertheless, its modest local up-regulation is also SA-dependent (Figure 3).

Increased expression of *PR1* upon SAR induction might directly contribute to resistance execution following fungal and oomycete pathogen attack, because PR1 proteins isolated from tobacco and tomato possess *in vitro* antifungal activity (Niderman et al., 1995). Moreover, overexpression of *PR1* in tobacco increases resistance to infection by the oomycetes *Peronospora tabacina* and *Phytophthora parasitica* var. *nicotianae* but has no protective effect on tobacco mosaic virus or *P. syringae* infection (Linthorst et al., 1989; Alexander et al., 1993; Rayapuram et al., 2008). Other SA-dependent SAR^+^ genes have a proven role in the activation of SA-associated defense responses that confers resistance to (hemi)biotrophic pathogens. For instance, the transcription factor WRKY70 has been recognized as a regulatory node that positively regulates SA-related plant defenses and suppresses JA-mediated responses. Overexpression of *WRKY70* increases basal resistance to *P. syringae* and to the powdery mildew *Erysiphe cichoracearum*, and results in the constitutive expression of SAR-related genes such as *PR1, PR2*, and *PR5*. Conversely, antisense suppression of *WRKY70* or insertional inactivation leads to enhanced expression of JA-responsive genes and compromises *E*. *cichoracearum* resistance (Li et al., 2004, 2006). In addition, *WRKY70* acts in concert with *WRKY53* and *WRK46*, two other SAR^+^ genes that belong to the SA-dependent and the partial SA-dependent gene cluster, respectively (Figures 3, 5). This is reflected by the finding that *wrky46/53/70* triple but not *wrky70* single mutants exhibit attenuated basal resistance towards *P. syringae* (Hu et al., 2012). Another SA-regulated SAR component that positive regulates *PR1* expression and is required for full basal resistance to *P. syringae* is the short chain dehydrogenase/reductase SDR3. The metabolic function of SDR3 has not been elucidated yet (Hwang et al., 2012). Moreover, NUDX6, a member of the Nudix (nucleoside diphosphate linked to moiety X) hydrolase family that catalyze the hydrolysis of several nucleoside diphosphate derivatives, not only acts in NADH metabolism in response to SA but also positively regulate SA-related defense responses (Ishikawa et al., 2010). *MILDEW RESISTANCE LOCUS O 2* (*MLO2*) belongs to a plant-specific family of genes coding for membrane proteins that contain seven transmembrane domains. *MLO2* contributes to Arabidopsis resistance towards attack by the necrotrophic fungus *Botrytis cinerea* and participates together with other components to non-host resistance of Arabidopsis to the rice blast pathogen *Magnaporthe oryzae* (Humphry et al., 2010; Nakao et al., 2011). Interestingly, MLO2 has been recognized as target of the *P. syringae* type III effector HopZ2 which physically interacts with MLO2. A *mlo2* insertion line exhibits increased resistance to *P. syringae*, suggesting that the MLO2/HopZ2-interaction is required for HopZ2-associated virulence (Lewis et al., 2012).

*NUDX6* and *GRXS13* are two examples of SA-dependent SAR^+^ genes that appear to function in redox homeostasis during SAR. *GRXS13* codes for a plant glutaredoxin which facilitates infection of Arabidopsis by *B. cinerea* (La Camera et al., 2011). Moreover, *GRXS13* expression is critical to limit basal and photooxidative stress-induced ROS production (Laporte et al., 2012). A redox-related function might also exist for At4g39830 which encodes a putative ascorbate oxidase (Yamamoto et al., 2005). Somewhat surprisingly, several SA-regulated SAR^+^ components obviously reduce SA accumulation and/or SA signaling and therefore appear to function in the containment of defense response activation during SAR establishment (Figure 3). For example, NIMIN-1 interacts with the SA receptor NPR1 in yeast-2-hybrid assays and functions as a negative regulator of SA-induced *PR1* expression (Weigel et al., 2005). The UDP-dependent glycosyltransferase UGT76B1 can glycosylate the Ile catabolite isoleucic acid and thereby negatively influences SA accumulation (von Saint Paul et al., 2011). Beyond that, UGT76B1 exhibits in vitro glycosylating activity towards SA, and conversion of free, signaling active SA to glycosylated derivatives is supposed to attenuate SA signaling (Noutoshi et al., 2012). Finally, the transcription factor WRKY38 negatively affects SA sensitivity and basal resistance to *P. syringae* (Kim et al., 2008).

Together, SA-dependent SAR^+^ genes can have distinct roles in the activation of defenses and resistance execution against different pathogen types (e.g. *PR1, WRKY70, WRKY53, SDR3, NUDX6, MLO2*), down-regulation of the JA pathway (*WRKY70*), redox homeostasis (*GRXS13, NUDX6*), and containment of defense response activation after SAR establishment (*NIMIN-1, UGT76B1, WRKY38*) (Figure 9). The specific functions of several other SA-regulated SAR^+^ genes still remain to be clarified.

### SAR^+^ genes that can be expressed independently of SA include critical SAR activators (SAR^+^ gene cluster II)

The group of SA-independent SAR^+^ genes contains at least three genes whose functions are necessary for SAR establishment: *ALD1, FMO1*, and *ICS1* (Figures 4, 9). ALD1 encodes a Lys aminotransferase (Song et al., 2004b) that mediates the biosynthesis of the Lys catabolite Pip, a critical SAR regulator (Návarová et al., 2012). Pip accumulation in 2° leaves of SAR-induced plants timely precedes SA accumulation, and Pip signaling requires the flavin-dependent monooxygenase FMO1 for SAR induction. The function of Pip as a metabolic amplifier of defense signaling is crucial for the ICS1-mediated accumulation of SA in 2° leaves (Mishina and Zeier, 2006; Návarová et al., 2012). Therefore, at the onset of SAR, SA-independent signaling events obviously trigger the expression of the key SAR^+^ genes *ALD1, FMO1*, and *ICS1* that are required for systemic SA accumulation (Figure 9). *ICS1* can be regarded as a “bridge” between SA-independent and SA-dependent SAR signaling events because its up-regulation results in the de novo biosynthesis of SA (Wildermuth et al., 2001). It is important to note that a feedback amplification mechanism in 2° leaves that involves ALD1, Pip, FMO1, ICS1, SA, and NPR1 exists to ensure full SAR establishment (Návarová et al., 2012; Shah and Zeier, 2013). Therefore, in the context of SAR signaling and establishment in 2° leaf tissue, SA-independent and SA-dependent signaling process cannot be regarded as separately acting unities. For instance, although the SA-deficient *sid2* mutant is able to accumulate wild-type like Pip levels in 1° leaves upon *P. syringae* inoculation, it accumulates markedly reduced Pip levels in 2° leaves (Návarová et al., 2012). Thus, Pip accumulation does occur independently from the capacity of SA biosynthesis at inoculation sites, but SA synthesis is required within the above-mentioned amplification cycle for the full accumulation of Pip in systemic tissue (Figure 9). Similarly, the systemic up-regulation of SA-independent genes might be substantially reduced in *sid2*, as has previously been shown for *FMO1* (Mishina and Zeier, 2006). Thus, the classification of SAR^+^ genes as “SA-independent” is based on expression characteristics in 1° leaves (Table 6) and does not consider the necessity of SA production for SAR-associated signal amplification in 2° leaves.

Another SAR^+^ gene up-regulated at inoculation sites in an SA-independent fashion is *PBS3* (alias GH3.12, GDG1, and WIN3) (Figure 4). Null mutants of *pbs3* exhibit severe defects in the induction of local resistance to bacterial infection and are significantly but not fully compromised in SAR (Jagadeeswaran et al., 2007; Lee et al., 2007; Nobuta et al., 2007). *PBS3* acts upstream of SA in the induction of immune responses and encodes a GH3 acyl adenylase that is able to conjugate 4-substituted benzoic acid derivatives to amino acids in vitro (Okrent et al., 2009). *HSP90-1* and *HSP70* are SA-independent SAR^+^ genes strongly up-regulated by H_2_O_2_ (Figure 4). *HSP90-1* encodes a cytosolic isoform of the heat shock protein HSP90 that associates with the co-chaperones SGT1 and RAR1 to mediate the maturation of various nucleotide-binding domain and leucine-rich repeat containing (NLR)-type of resistance proteins. Gene knockouts of *RAR1, SGT1* or *HSP90* compromise resistance against various bacterial, fungal and viral pathogens (Kadota and Shirasu, 2012). Arabidopsis *SGT1a* but not *SGT1b* or *RAR1* falls into the category of (SA-dependent) SAR^+^ genes (Figure 9). HSP70 (alias HSC70-4) represents one of four cytosolic HSC70 isoforms that, similarly to HSP90, interact with the co-chaperone SGT1. Knockout of individual cytosolic *HSC70* genes has no defense phenotype, but *HSC70-1* overexpression compromises resistance to virulent and avirulent pathogens (Noël et al., 2007).

**Figure 8:**
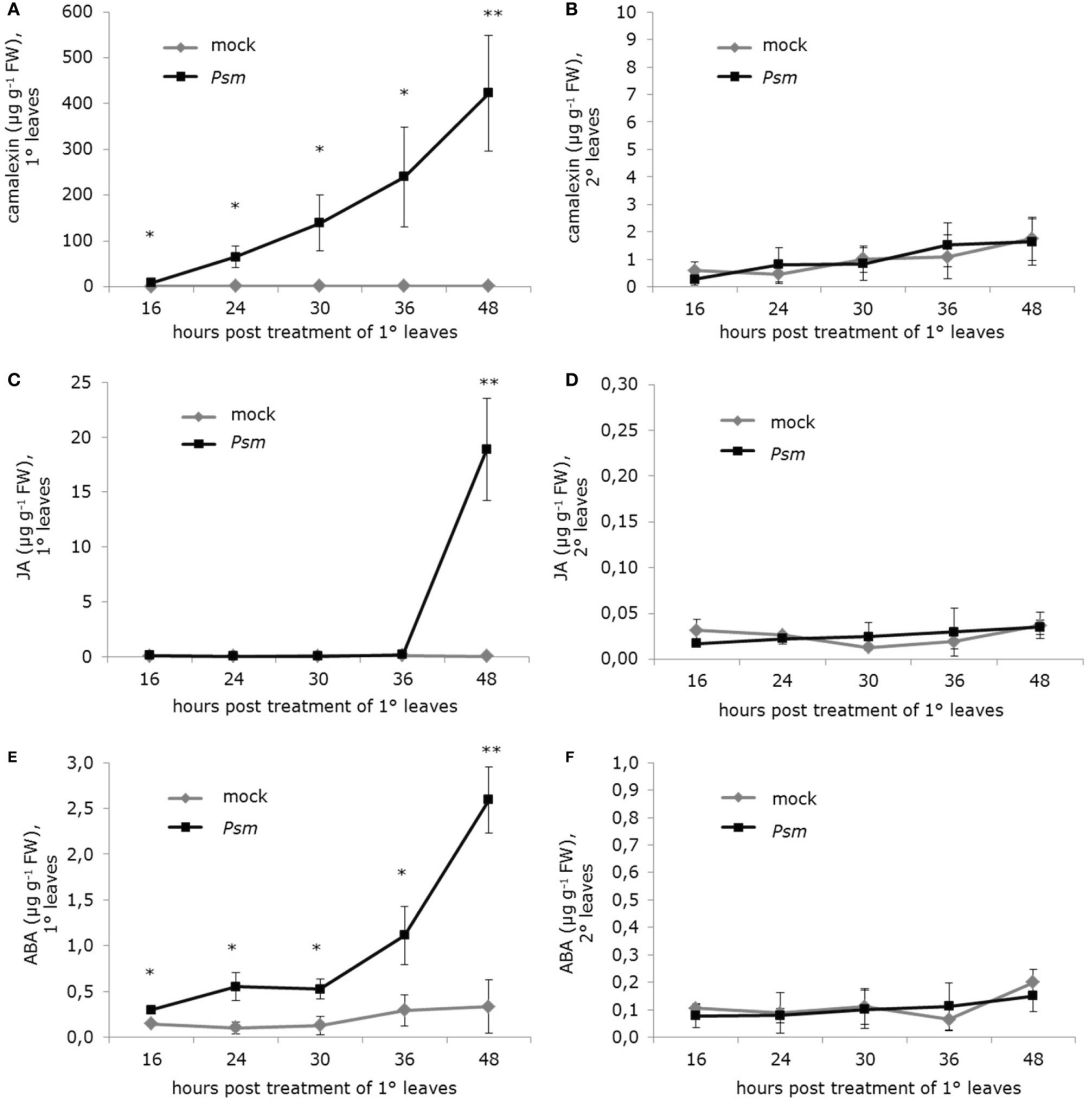
**Levels of camalexin, jasmonic acid (JA), and abscisic acid (ABA) in treated (1°) and non-treated distal (2°) leaves of Arabidopsis Col-0 plants inoculated with a suspension of *P. syringae* pv. *maculicola* (*Psm*; OD 0.005) or infiltrated with a 10 mM MgCl_2_ solution (mock-treatment)**. Data represent the mean ± SD of at least three replicate samples. Asterisks denote statistically significant differences between *Psm*- and mock-samples (^**^P < 0.01; ^*^P < 0.05; two-tailed *t*-test). **(A,B)** Camalexin levels at indicated times post treatment of 1° leaves in **(A)** 1° leaves and **(B)** 2° leaves. **(C,D)** JA levels at indicated times post treatment of 1° leaves in **(C)** 1° leaves and **(D)** 2° leaves. **(E)** and **(F)** ABA levels at indicated times post treatment of 1° leaves in **(E)** 1° leaves and **(F)** 2° leaves.

**Figure 9:**
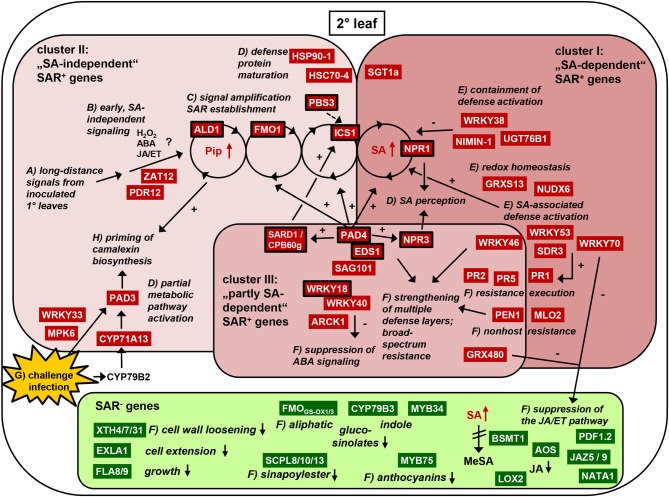
**Events occurring in distal (2°) leaves of Arabidopsis plants in which SAR has been biologically activated by *Psm* inoculation in 1° leaves**. SAR up-regulated (SAR^+^ genes) are depicted in red, SAR down-regulated genes (SAR^−^ genes) depicted in green. Genes known to be indispensable for SAR activation are framed with a black line. The three clusters of SA-independent, SA-dependent, and partially SA-dependent SAR^+^ genes are illustrated. Processes leading to SAR establishment and functions of individual SAR-related genes or groups of genes are italicized. The alphabetical labels indicate a hypothetical order of events. **(A)** First, long-distance signals derived from inoculated leaves activate initial SA-independent signaling events. **(B)** Possible contributions of ROS, ABA, or JA/ET to these initial events are hypothetic or even doubtful. **(C)** A feedback amplification cycle (depicted as interconnected wheels) that requires the accumulation and the action of the two critical SAR metabolites pipecolic acid (Pip) and salicylic acid (SA) as well as the function of the flavin-monooxygenase FMO1 establishes SAR. (**D–F)** Various events such as the activation of SA signaling, resistance induction, suppression of JA- and ABA-signaling, and partial pre-activation of camalexin biosynthesis occur. **(G,H)** SAR-induced plants are primed for early defense activation such as camalexin accumulation and defense gene expression. Small up arrows symbolize metabolite accumulation, small down arrows symbolize reduction of metabolite biosynthesis or of indicated physiological responses. Large arrows indicate the interconnection between the responses. Plus-signs symbolize activation, minus-sings repression. The indicated events are described in detail in the main text and summarized in the “Summary and conclusions” paragraph.

A typical local response of Arabidopis leaves to infection with necrotrophic or (hemi)biotrophic pathogens is the accumulation of the indolic phytoalexin camalexin (Glawischnig, 2007). In *Psm*-inoculated plants, camalexin is heavily produced in locally infected tissue but the phytoalexin does not accumulate systemically in 2° leaves (Figures 8A,B). However, SAR-induced plants are conditioned to more vigorously synthesize camalexin in response to subsequent pathogen encounter, and this priming effect is mediated by Pip (Návarová et al., 2012). The induction of camalexin biosynthesis at pathogen inoculation sites is associated with a modest activation of genes coding for enzymes of the Trp biosynthetic pathway and strong induction of genes encoding cytochrome P450 monooxygenases involved in Trp catabolism towards camalexin: *CYP79B2/3, CYP71A13*, and *CYP71B15* (alias *PAD3*) (Ren et al., 2008). In 2° leaves of SAR-induced plants, *PAD3* expression is strongly and *CYP71A13* expression is moderately up-regulated, and both genes belong to the SA-independent cluster (Figure 4). By contrast, *Psm*-inoculation does only lead to a local but not a systemic up-regulation of *CYP79B2* (Figure 7), and *CYP79B3* is neither locally nor systemically up-regulated. This expression pattern is consistent with the observed lack of systemic camalexin accumulation because *CYP79B* expression and thus the metabolic step from Trp to indole-3-acetaldoxime (IAOx) are not activated in 2° leaves of SAR-induced plants. However, enhanced systemic expression of *CYP71A13* and *PAD3* pre-activates the pathway downstream of IAOx. This partial biosynthetic pathway activation can explain why camalexin accumulation is primed upon SAR induction and therefore can occur faster and more pronouncedly in challenged SAR-induced than in challenged control plants: key enzymes of the pathway are already expressed before the challenge inoculation takes place, and the ab initio expression of fewer components (for camalexin biosynthesis probably merely CYP79B2) is therefore required to provide the full enzymatic capacity for the biosynthesis of the metabolite (Figure 9). The WRK33 transcription factor controls the activation of camalexin biosynthetic genes and camalexin production, and WRKY33 activity is regulated via a MAP kinase cascade involving MPK3 and MPK6 (Mao et al., 2011). *WRKY33* and *MPK3* but not *MPK6* are up-regulated during SAR in a largely SA-independent manner (Figures 4, 5).

As stated above, the average responsiveness to H_2_O_2_, ABA, and JA/ET is higher for SAR^+^ genes assigned to the SA-independent cluster than for those assigned to the SA-dependent cluster (Figure 2B). This is based on the fact that individual SA-independent genes are strongly inducible by H_2_O_2_ (e.g. *CYP81D8, WRKY33, BCB, HSP70, HSP90-1, ZAT12*) or ABA (e.g. *CHI, SAG13, GST22, HSP70*), and that some genes are modestly inducible by JA/ET signaling (e.g. *AIG2, GST22, PDR12, FMO1*). Moreover, *PDR12* encodes an ATP-binding cassette (ABC) transporter that mediates cellular uptake of ABA (Kang et al., 2010), and *ZAT12* codes for a transcription factor implicated in ROS signaling (Davletova et al., 2005). This raises the question whether an early wave of ROS-, JA/ET-, and/or ABA-signaling would precede Pip- and SA-mediated establishment of SAR in 2° leaves (Figure 9). An earlier work on *P. syringae-induced* SAR has reported the involvement of systemic ROS micro-bursts in the SAR regulatory network of 2° leaf tissue that requires an early oxidative burst in 1° inoculated leaves. These systemic microbursts were only observed in plants inoculated with avirulent but not with virulent *P. syringae* (Alvarez et al., 1998). However, the fact that both avirulent and virulent *P. syringae* are able to induce a typical SAR response argues against a critical role of microbursts in SAR activation (Mishina and Zeier, 2006, 2007; Attaran et al., 2009; Jing et al., 2011; Liu et al., 2011). Moreover, the occurrence of systemic microbursts during SAR has not been confirmed by other studies. However, wounding and different abiotic stresses can trigger systemic ROS signaling that is dependent on the NADPH oxidase RBOHD (Miller et al., 2009). Miller et al., reported that the RBOHD-dependent signal can translocate from wounded to systemic tissue within minutes. This rapid systemic distribution of the ROS-related signal is not in accordance with the relatively slow establishment of SAR that essentially takes place between day 1 and day 2 after 1° pathogen inoculation for the *Psm*-Arabidopsis interaction (Mishina et al., 2008), and it is thus not clear whether ROS signaling indeed contributes to SAR establishment.

Is the relatively high number of strongly ABA- and moderately JA/ET-responsive genes in the SA-independent group indicative for early ABA- and JA/ET-signaling events required for SAR establishment? Truman et al. (2007) have described an early JA response in 2° leaves of plants inoculated with very high titers [OD_600_ = 0.2] of avirulent *P. syringae* pv. *tomato* DC3000 (*Pst*) expressing the *avrRpm1* avirulence gene (*Pst avrRpm1*) that was associated with an increase of JA in petiole exudates collected from 1° leaves. These high initial titers of *Pst avrRpm1* induce a rapid HR in leaves, and the necrotic disruption of leaf tissue goes hand in hand with the accumulation of JA and other oxylipins (Mishina and Zeier, 2007; Zoeller et al., 2012). By contrast, our experimental conditions include bacterial inoculations with lower densities (OD_600_ = 0.005) of compatible *Psm*, which are not associated with an HR and elevations of JA in petiole exudates (Návarová et al., 2012). Although JA levels rise in *Psm*-inoculated leaves at about 48 hpi, systemic rises of JA are not detected between 16 to 48 hpi (Figures 8C,D). Considering these data and the above-mentioned 24 hpi to 48 hpi time window in which SAR establishment takes place in *Psm*-inoculated Arabidopsis plants, it seems unlikely that JA signaling contributes to the up-regulation of the moderately JA/ET-responsive SAR^+^ genes of the SA-independent gene cluster. Irrespective of the existence of an early wave of systemic JA signaling after bacterial inoculation, its influence on SAR induction is supposedly negligible, because different Arabidopsis mutants compromised in JA biosynthesis or downstream signaling are SAR-competent (Attaran et al., 2009).

The accumulation of ABA in *Psm*-inoculated leaves occurs more gradually and faster than the accumulation of JA (Figure 8E), and the kinetics of ABA production in leaves upon bacterial attack is similar for *Psm*- and *Pst*-inoculations (Figure 8E, Fan et al., 2009). Again, the accumulation of ABA, which negatively influences SA signaling and counteracts SAR (Fan et al., 2009), is confined to the site of bacterial inoculation and does not occur in 2° leaf tissue (Figure 8F). This metabolic data argues against a function for ABA as an initial trigger for the expression of SA-independent SAR^+^ genes in 2° leaves during SAR.

### The SAR^+^ gene cluster III contains genes exhibiting partly SA-dependent expression

The third cluster of SAR up-regulated genes contains genes that are essential for the initiation of the SAR process as well (Figures 5, 9). These include *PAD4, ENHANCED DISEASE SUSCEPTIBILITY 1* (*EDS1*), *SARD1, CBP60g*, and *NPR3* (Mishina and Zeier, 2007; Truman et al., 2007; Zhang et al., 2010; Rietz et al., 2011; Fu et al., 2012). These genes are less tightly regulated by SA than the cluster I genes (Figure 3), because, unlike the latter, their [S/M]_Psm_ ratios are (per definition) at least 1.5-fold higher than the corresponding [Col/*sid2*]_Psm_ values (Figure 5). PAD4, EDS1, and another SAR^+^ gene product from cluster III, SAG101 (Figure 5), constitute a family of plant-specific hydrolase proteins that are critical regulatory components of plant basal resistance to (hemi)biotrophic pathogens and ETI triggered by a subset of resistance proteins (Wiermer et al., 2005). Apart from forming homodimers, EDS1 can interact with PAD4 and SAG101, and the formation of different EDS1 homo- or heteromeric complexes is associated with distinct localization patterns in the cytoplasm and/or nucleus (Feys et al., 2005). Complex formation between EDS1 and PAD4 is required for the full establishment of SAR (Rietz et al., 2011). Moreover, the EDS1-PAD4-SAG101 signaling complex also plays an important function in Arabidopsis post-invasion nonhost resistance to non-adapted powdery mildew fungi (Lipka et al., 2005). The [Col/*pad4*]_Psm_ ratios illustrate that expression of the predominant fractions of SAR^+^ genes from all three distinguished clusters is more or less tightly regulated by PAD4, indicating an important function for PAD4 in overall SAR^+^ gene transcription (Figures 2B, 3, 4, 5, 9). For instance, the expression of the critical SAR regulatory pathway genes *ALD1, FMO1*, and *ICS1* are all partially dependent on PAD4 (Figure 5; Song et al., 2004a), and consequently, accumulation of the SAR regulatory metabolites Pip and SA are both positively regulated by PAD4 (Zhou et al., 1998; Návarová et al., 2012). Moreover, PAD4 tightly regulates the *Psm*-induced expression of two members of the plant-specific transcription factor gene family ACBP60, *SAR-DEFICIENT1* (*SARD1*) and *CALMODULIN-BINDING PROTEIN60G (CBP60g)*, as well as the *NPR1* homologue *NPR3* (Figure 5). The SARD1 and CBP60g transcription factors are partly redundant in their function and activate pathogen-induced *ICS1* transcription resulting in SA accumulation (Zhang et al., 2010; Wang et al., 2011). A *sard1 cbpg60g* double mutant is therefore completely SAR defective (Zhang et al., 2010). NPR3 has been recently identified, besides NPR1 and NPR4, as a bona fide SA receptor (Fu et al., 2012; Wu et al., 2012). Therefore, the SAR^+^ gene cluster III contains central elements of both SA biosynthesis and SA downstream signaling.

The SAR^+^ gene cluster III also contains the two WRKY transcription factor genes *WRKY18* and *WRKY40* (Figure 5). WRKY18, WRKY40 and WRKY60 (*WRKY60* is not a SAR^+^ gene) constitute a group of sequence-related WRKYs with complex and partly redundant roles in plant defense against different pathogen types. WRKY18 is required for biological SAR activation and mediates a subset of NPR1-mediated responses (Wang et al., 2006). Whereas overexpression of *WRKY18* alone increases resistance to *P. syringae*, simultaneous overexpression of *WRKY18* and *WRKY40* enhances susceptibility to the same pathogen (Xu et al., 2006). WRKY18/40/60 negatively regulate ABA signaling (Shang et al., 2010), and ABA signal transduction is also attenuated by *ARCK1*, another cluster III SAR^+^ gene that encodes a receptor-like cytosolic protein kinase (Tanaka et al., 2012). Since ABA signaling can interfere with the SA pathway and thus attenuate plant defenses against (hemi)biotrophic pathogens (de Torres-Zabala et al., 2007; Fan et al., 2009), an impairment of the ABA pathway might ensure a robust SA response during SAR (Figure 9). Another cluster III SAR^+^ gene is *GRX480* encoding for a glutaredoxin that interacts with TGA transcription factors and negatively affects JA and ET signaling. This results in the suppression of expression of typically JA/ET-regulated genes such as *PDF1.2* (Ndamukong et al., 2007; Zander et al., 2012).

The group III also contains the two SAR marker genes *PR2* and *PR5* which, similar to *PR1*, may function in the direct execution of disease resistance because their gene products exhibit antimicrobial activity. Plant *PR2* genes code for β-1,3-glucanases, and purified β-1,3-glucanases from pea have been shown to act synergistically with chitinases in the degradation of fungal cell walls (Mauch et al., 1988). The PR5 protein family includes the basic osmotins whose members are homologous to the sweet-tasting protein thaumatin. Osmotin and other *PR5* proteins have been shown to exhibit antifungal activity in vitro and in planta. For example, overexpression of tobacco osmotin in different plant species results in increased resistance to oomycete pathogens of the genus *Phytophtora* (Liu et al., 1994). Moreover, SAR^+^ group III contains *PEN1*, a critical determinant of Arabidopsis pre-invasion nonhost resistance to non-adapted powdery mildew fungi. *PEN1* codes for a plasma membrane-resident syntaxin which becomes recruited at sites of attempted fungal ingress and is implicated in a vesicle-associated resistance mechanism that prevents fungal penetration through epidermal cell walls (Collins et al., 2003; Bhat et al., 2005). The up-regulation of genes involved in non-host resistance, basal resistance to different pathogen types and ETI indicate that SAR simultaneously strengthens different defense layers that make up the plant immune system (Thordal-Christensen, 2003).

### A significant portion of the genes down-regulated during SAR exhibit strong JA-responsiveness

A remarkable feature of the SAR^−^ genes is that their average expression is markedly activated via the JA signaling pathway (mean [Col/*coi1*]_Psm_ = 5.6; Figure 2A). Taken the [Col/*coi1*]_Psm_ values as a basis, we divided the SAR^−^ genes into three categories: JA-activated genes ([Col/*coi1*]_Psm_ > 2; 76 genes), JA-repressed genes ([Col/*coi1*]_Psm_ < 0.5; 190 genes), and JA-independent genes ([Col/*coi1*]_Psm_ > 0.5 and < 2; 404 genes) (Figure 2C). Although quantitatively the smallest group, the JA-activated genes most strongly influenced the average [Col/*coi1*]_Psm_ ratio because most genes in this group have very high [Col/*coi1*]_Psm_ ratios (average [Col/*coi1*]_Psm_ = 43.9). Apart from a few exceptions (e.g. *PDF1.2*), this is associated with high [S/M]_JA_ ratios (average [S/M]_JA_ = 10.3) (Figures 2C, 6). Thus, both the [Col/*coi1*]_Psm_ and the [S/M]_JA_ ratios indicate that the members of the JA-activated group of SAR^−^ genes are highly responsive to JA signaling.

In fact, the JA-activated group of SAR^−^ genes consist of a series of genes typically regarded as marker genes of the JA pathway (Figure 6). Among them are *JASMONATE-REGULATED 21* (*JRG21*) (Nickstadt et al., 2004), *JASMONATE ZIM-DOMAIN PROTEIN 5* (*JAZ5*), *JAZ9* (Thines et al., 2007), *BENZOIC ACID/SA CARBOXYL METHYLTRANSFERASE 1* (*BSMT1*) (Chen et al., 2003), *N-ACETYLTRANSFERASE ACTIVITY 1* (*NATA1*) (Adio et al., 2011), *CORONATINE-INDUCED PROTEIN 1* (*COR1*) (Benedetti et al., 1998), *JASMONIC ACID RESPONSIVE 1* (*JR1*), *JR2* (León et al., 1998), *POLYGALACTURONASE INHIBITING PROTEIN 2* (*PGIP2*) (Schenk et al., 2003), *ALLENE OXIDE SYNTHASE* (*AOS*) (Laudert and Weiler, 1998), *LIPOXYGENASE 2* (*LOX2*) (Bell and Mullet, 1993), and the plant defensin *PDF1.2* (Ndamukong et al., 2007). This indicates that JA defense signaling is significantly reduced in the SAR-induced state. One of the key genes involved in JA biosynthesis, *AOS* (Laudert and Weiler, 1998), is markedly down-regulated during SAR (Figure 6), suggesting that the JA pathway could be attenuated already at the level of JA biosynthesis. However, since the experimentally determined levels of JA are similarly low in 2° leaves of mock-control and *Psm*-inoculated plants (Figure 8D), it is more likely that signaling events downstream of JA production are negatively affected during SAR. SAR is characterized by activated SA signaling (Figure 2A), and the well-established negative cross-talk between the SA- and JA-pathways might be responsible for the attenuation of JA responses (Spoel et al., 2003). As discussed above, molecular players such as the SA-activated transcription factor WRKY70 and the glutaredoxin GRX480 could mediate the suppression of the JA pathway during SAR (Li et al., 2004, 2006; Ndamukong et al., 2007) (Figure 9). Moreover, the decreased expression of the SA methyltransferase BSMT1 that converts signaling active SA into inactive methyl salicylate (MeSA) supposedly counteracts deactivation of the SA signal (Attaran et al., 2009; Wu et al., 2012) (Figure 9). SAR is associated with a reduced biosynthesis of constitutively produced metabolites such as glucosinolates or sinapoylmalates, as illustrated by the down-regulation of *MYB34*, a JA-inducible transcription factor that activates indolic glucosinolate production (Figures 6, 9) and by the decreased expression of several sinapoyltransferase genes (Table 5). Another group of secondary metabolites whose biosynthesis might be negatively affected upon SAR activation are anthocyanins, because *MYB75* involved in the transcriptional regulation of anthocyanin biosynthesis (Borevitz et al., 2000) also belongs to the group of SAR-repressed and JA-activated genes (Figures 6, 9). It is likely that the production of inducible metabolites that negatively interfere with resistance to (hemi)biotrophic pathogens is also repressed during SAR. For instance, the JA-inducible acetyltransferase NATA1 mediates the formation of N-δ-acetylornithine from ornithine. Since *nata1* knockout lines are more resistant to *P. syringae* than the wild-type, N-δ-acetylornithine is supposed to negatively influence bacterial resistance (Adio et al., 2011). A reduced induction of *NATA1* expression during SAR (Figure 6) could therefore counteract the presumed negative effect of N-δ-acetylornithine on bacterial resistance. This would be consistent with the finding of Adio et al. (2011) that SA pre-treatment, which renders plants in an enhanced state of pathogen resistance, inhibits induced N-δ-acetylornithine formation.

### Similarities and differences of defense activation in 1° and 2° leaf tissue upon bacterial inoculation

The signaling network underlying basal resistance to local infection exhibits overlapping features to the signaling events that activate SAR because both forms of resistance share similar regulatory factors such as ICS1, SA, NPR1, PAD4, EDS1, ALD1, Pip, and FMO1. ALD1-mediated Pip production and FMO1-dependent transduction of Pip signaling do occur in both *P. syringae*-inoculated and in systemic leaf tissue (Návarová et al., 2012). Pip functions as a mediator of defense amplification in plants, and this fortification of defense responses is indispensable for the activation of SAR (Návarová et al., 2012; Shah and Zeier, 2013). The Pip/FMO1-resistance pathway is also important for local resistance induction but the extent of its impact on basal resistance appears to vary with the attacking pathogen type (Song et al., 2004a; Bartsch et al., 2006; Mishina and Zeier, 2006; Návarová et al., 2012). Further, SA accumulation and downstream signaling are common processes induced in 1°-inoculated leaf tissue and in distant 2°-leaves, and the activation of the SA pathway is required for both basal resistance to (hemi)biotrophic pathogens and SAR (Wildermuth et al., 2001; Mishina and Zeier, 2006; Spoel and Dong, 2012). The existence of common immune regulatory metabolites in 1° and 2° leaf tissue and the fact that most SAR^+^ genes are also up-regulated in inoculated tissue after pathogen encounter illustrates that overlapping signaling principles and defense mechanisms exist in inoculated 1° and in systemic 2° leaves (Figures 2A,B, 3, 4, 5).

Are there characteristic differences at the levels of defense metabolite production and gene activation in 1° and 2° leaf tissue? A first difference is of quantitative nature: Pip and SA accumulate to markedly higher levels in 1° than in 2° leaf tissue (Mishina et al., 2008; Návarová et al., 2012). In addition, the levels of a significant higher number of metabolites increase in 1° than in 2° leaf tissue after *P. syringae* inoculation (Ward et al., 2010; Griebel and Zeier, 2010; Chanda et al., 2011; Návarová et al., 2012). As shown in Figure 8, the substances that strongly accumulate in 1° but not in 2° leaf tissue include JA, ABA, and camalexin. In addition, when comparing the number of genes up- or down-regulated in 1° and 2° leaves of *Psm*-treated plants in different experiments, it becomes obvious that the transcriptional changes at inoculation sites are much more pronounced than those in distant tissue (Figure 2D). From the 1921 Arabidopsis genes assigned to be locally up-regulated at 24 h post *Psm*-inoculation (Figure 2D), 299 and 19 belong to the groups of SAR^+^ and SAR^−^ genes, respectively. This implies that the expression levels of about 15.5 % of genes locally up-regulated at 24 hpi do increase systemically after SAR establishment, but that the expression of the largest portion of locally induced genes essentially remains unchanged at the systemic level. Strikingly, the average [Col/*coi1*]_Psm_ ratio in the group of locally up-regulated genes is high (6.4) compared to the same value for 2° leaves (1.1) (Figure 2D), indicating that JA signaling is strongly activated in 1° but not in 2° leaf tissue, in which, as discussed in the previous section (Figures 2C, 6), the expression of many JA-responsive genes is even reduced. The stimulation of JA signaling in 1° leaves can be triggered by the *P. syringae* phytotoxin coronatine, a structural mimic of the signaling active JA derivative JA-Ile (Geng et al., 2012), and by endogenously produced JA. In phases of the plant-bacterial interaction during which endogenous JA levels remain low (e.g. until 36 hpi for the *Psm*-Arabidopsis inoculation experiment shown in Figure 8C), bacterial coronatine is presumably the major stimulus. For instance, Attaran et al. (2009) performed a comparative assessment of the formation of MeSA in Arabidopsis leaves induced by coronatine-producing and non-producing *Pst*. MeSA is generated by the BSMT1-catalysed methylation of SA, and the *BSMT1* gene is strongly JA-responsive (Figure 7). Until 24 hpi, only the coronatine-producing but not the coronatine- deficient *Pst* strain elicited a significant formation of MeSA in inoculated plants, indicating that bacterial-derived coronatine rather than endogenous JA triggers the metabolic response in earlier phases of the interaction (Attaran et al., 2009).

The strong activation of the JA pathway in inoculated leaves and the partial suppression of JA responses in distant leaves reflect a major difference between the hormonal status of 1° and 2° tissue, and this difference impacts the nature of defense responses in both tissue types. JA pathway activation at inoculation sites is at least partially causative for the more pronounced metabolite accumulation in 1° compared to 2° leaves because JA signaling induces the expression of a series of genes involved in metabolite biosynthesis. For example, the biosynthesis of many mono-, sesqui- and diterpenoids in plants is characteristically regulated via JA signaling (Arimura et al., 2008; Attaran et al., 2008). Indeed, among the most strongly up-regulated genes in *Psm*-inoculated tissue are JA-inducible genes involved in metabolism such as the SA methyl transferase *BSMT1*, the terpene synthase *TPS4*, several cytochrome P450 monooxygenases (e.g. *CYP82G1, CYP94C1, CYP94B3, CYP79B2*), and UDP-dependent glycosyltransferases (*UGT76E12, UGT76E1*) (Figure 7). *P. syringae*-inoculated Arabidopsis leaves produce the C_16−_homoterpene (E,E)-4,8,12-trimethyl-1,3,7,11-tridecatetraene (TMTT) in a *TPS4*-dependent manner (Attaran et al., 2008). TPS4 catalyses the first step of TMTT biosynthesis, i.e. the conversion of the diterpene precursor geranylgeranyl diphosphate to (E,E)-geranyllinalool (Herde et al., 2008). Subsequent formation of the C_16_-homoterpene from (E,E)-geranyllinalool by an oxidative cleavage reaction catalyzed by CYP82G1 completes TMTT biosynthesis (Lee et al., 2010). The two cytochrome P450 enzymes CYP94B3 and CYP94C1 are involved in the catabolic turnover of the signaling active jasmonate JA-Ile. CYP94B3 mediates the hydroxylation of JA-Ile to 12-hydroxy-JA-Ile and thereby inactivates hormone function (Koo et al., 2011). CYP94C1 then converts 12hydroxy-JA-Ile to the corresponding 12-carboxy-derivative (Heitz et al., 2012). Another strongly *Psm*-up-regulated gene whose expression is only moderately affected by the JA signaling pathway is *1-AMINO-CYCLOPROPANE-1-CARBOX-YLATE SYNTHASE 2 (ACS2)*, an ACS isoform involved in ethylene biosynthesis (Tsuchisaka et al., 2009). The activation of ET biosynthesis in *P. syringae*-inoculated leaves is consistent with the microarray gene expression data, because ET-dependent genes are expressed more prominently in 1° (mean [Col/*ein2*]_Psm_ = 1.7) than in 2° (mean [Col/*ein2*]_Psm_ = 1.1) leaves (Figures 2D, 7). Examples of genes partially regulated via ET signaling are *ALPHA-DIOXYGENASE 1* (*DOX1*), encoding a fatty acid α-dioxygenases which converts linolenic acid and other fatty acids into their 2-hydroperoxy derivatives (Hamberg et al., 1999). Moreover, *PR3* encodes a basic chitinase that is up-regulated in 1° inoculated but not in 2° tissue and regulated by JA/ET signaling (Figure 7; Zander et al., 2010).

Another obvious difference between the transcriptional changes in 1° and 2° leaves following *Psm* inoculation is the stronger activation of ABA-responsive genes at inoculation sites compared to systemic tissue (mean [S/M]_ABA_ = 3.3 and 1.5 for 1° and 2° leaves, respectively; Figures 2D, 7), which is consistent with the observation that ABA accumulation is restricted to bacterial inoculation sites (Figure 8E). The [S/M]_ABA_ values indicate that ABA signaling in 1° leaves contributes to the induction of genes such as *ANAC019, DOX1, CYP94B3, UGT74E2*, and *CYP710A1* (Figure 7). The transcription factor ANAC019 binds to a drought-responsive cis-element in the *early responsive to dehydration stress 1* promoter, and overexpression of *ANAC019* in Arabidopsis provides increased drought tolerance (Tran et al., 2004). This is one example of the fact that the local transcriptional responses following compatible *P. syringae* inoculation show large overlap with those occurring after drought or osmotic stress (de Torres-Zabala et al., 2007), presumably because strong bacterial proliferation in leaf tissue is associated with water deprivation and tissue necrosis. Activation of ABA signaling in infected tissue has also been interpreted as an active, effector-triggered virulence strategy of the pathogen, because ABA negatively interferes with SA signaling and therefore weakens one of the major pathways of plant defense to (hemi)biotrophic pathogens (de Torres-Zabala et al., 2007; Fan et al., 2009). Moreover, the ABA- and H_2_O_2_-responsive UDP-depedent glucosyltransferase *UGT74E2* that is also strongly up-regulated at inoculation sites has been implicated with the modulation of water stress responses. UGT74E2 glycosylates indole-3-butyric acid and therefore affects auxin homeostasis in plants (Tognetti et al., 2010), and modulated auxin signaling can result in disturbed plant immune responses (Truman et al., 2010). The pathogen-induced *CYP710A1* gene is also moderately ABA- and H_2_O_2_-responsive (Figure 7), and CYP710A1 mediates the desaturation of the most common phytosterol in Arabidopsis, β-sitosterol, to produce sitosterol (Morikawa et al., 2006), which strongly accumulates in *P. syringae*-inoculated leaves, integrates into cell plasma membranes, and negatively affects plant defense and resistance to bacteria (Griebel and Zeier, 2010).

## Summary and conclusions

Based on gene expression data, metabolite data, and literature information, the present study aimed to contribute to a better understanding of the characteristics of the SAR-induced state in plants. Figure 9 summarizes main events occurring in 2° leaves of plants after biological SAR activation. These include:

1. The establishment of SAR in Arabidopsis in response to a localized leaf inoculation with the bacterial pathogen *Psm* is associated with a major transcriptional reprogramming in distant leaf tissue. Thereby, several hundred genes that are systemically up- (SAR^+^ genes) and down-regulated (SAR^−^ genes) can be distinguished. This extensive transcriptional reprogramming upon SAR induction is dependent on the SAR regulatory gene *FMO1*.
2. Functional categorization on the basis of GO annotations indicates that the SAR^+^ gene cluster is enriched in genes associated with stress responses, signal transduction, transport, and the cell secretory apparatus, whereas in the SAR^−^ gene cluster, genes associated with the chloroplast, cell wall loosening, cell extension, and the biosynthesis of constitutively formed secondary metabolites are over-represented. This suggests that, upon SAR induction, plants redirect some of their resources from vegetative growth towards defense-related processes.
3. Alignment of the SAR expression data with publicly available microarray information has allowed us to classify the SAR-associated genes and analyse their expression characteristics. However, since the microarray data compared in our study originate from different laboratories, experimental parameters such as plant age, growth conditions, the kind of treatment or the timing of sample collection varied between experiments (Tab. 6). For instance, whereas soil grown, 4 to 5 week-old Arabidopsis plants were used for the *P. syringae*-, the flg22-, the SA-, and BTH-treatments, 5-7 day-old seedlings grown on MS medium were used for the JA-, ABA-, and H_2_O_2_-treatments. Although these experimental differences might have impact on the expression characteristics of individual genes, the predominant part of gene regulatory principles described in this work appears robust. This is exemplified by the findings that the JA- or SA-inducibility of genes was inferred from mutant analyses ([Col/*coi1*]_Psm_ or [Col/*sid2*]_Psm_) and exogenous treatment ([S/M]_JA_ or [S/M]_SA_) with considerable conformity (Figures 3, 6). Moreover, the stimulus-dependent regulation of the (SAR-related) genes discussed in this study proved consistent with available literature data.
4. Based on the expression patterns of SAR-related genes in *Psm*-inoculated wild-type and *sid2/ics1* leaves and on the responsiveness of those genes to exogenous SA, we have categorized the group of SAR^+^ genes into clusters of SA-independent genes (cluster II), SA-dependent genes (cluster I), and partially SA-dependent genes (cluster III).
5. Albeit not congruent, extensive similarities of the transcriptional responses of Arabidopsis plants following biological SAR induction and treatment with the synthetic resistance activator BTH do exist. The so-called “SA analogue” BTH exhibits a broader effect on SAR-related gene expression than the endogenous defense signal SA.
6. The cluster of SA-independent SAR^+^ genes contains the three critical SAR components *ALD1, FMO1*, and *ICS1*, which are indispensable for SAR establishment. *ALD1* and *FMO1* are required for the biosynthesis and downstream signaling, respectively, of the immune regulator pipecolic acid, which mediates SAR activation via signal amplification (Návarová et al., 2012). *ICS1* is involved in the de novo biosynthesis of SA (Wildermuth et al., 2001) and ”connects” the SA-independent and SA-dependent phases of SAR.
7. In the initial stages of SAR establishment in 2° leaves, SA-independent signaling might precede and then activate an SA-dependent phase of SAR. Since both phases are required for the full activation of SAR, “SA-independent” and “SA-dependent” signaling events cannot be regarded as separately acting units but tightly co-operate to realize SAR. The average responsiveness to H_2_O_2_, ABA, and JA/ET is higher for SAR^+^ genes from the SA-independent than for those from the SA-dependent group. This might suggest a role of these stimuli in the early signaling stages of SAR in 2° leaves. However, metabolite and mutant analyses rather argue against this possibility.
8. On average, SA-dependent SAR^+^ genes exhibit a higher responsiveness to flg22 than SA-independent SAR^+^ genes. (Partially) SA-dependent SAR^+^ genes function in the activation or maintenance of distinct defense layers (non-host resistance, basal resistance, ETI), and in resistance execution against different pathogen types. This indicates that SAR heightens the plant immune system on several levels and illustrates the hallmark of SAR as a state of broad-spectrum resistance. Other SA-regulated SAR^+^ genes are involved in redox homeostasis and in the containment of defense response activation after SAR establishment.
9. Negative cross-talk between JA/ET- or ABA-signaling pathways on the SA defense pathway is well-documented (Spoel et al., 2003; Fan et al., 2009). Several SAR^+^ genes are involved in the suppression of ABA- and JA/ET-signaling, suggesting that they can relieve inhibitory effects of these hormonal pathways on the SA pathway during SAR. Suppression of JA signaling during SAR also manifests itself in the fact that many highly JA-responsive genes are among the most strongly down-regulated genes during SAR.
10. Overlapping defense principles exist for the induction of local resistance responses and SAR. However, the transcriptional and metabolic responses at sites of bacterial inoculation are generally more pronounced than those in systemic tissue. A major difference between the 1° inoculated and the 2° leaves relates to the stress hormonal status: whereas SA, Pip, JA, and ABA are produced at inoculation sites to largely high levels, only Pip and SA moderately accumulate at the systemic level (Figure 8; Mishina et al., 2008; Návarová et al., 2012). Therefore, JA/ET- and ABA-triggered responses are strongly induced in 1° leaves, whereas these responses are not activated or even suppressed (see above) in the 2° leaves.
11. Pip accumulation during SAR primes plants to more quickly and effectively activate defense responses to subsequent pathogen encounter. A strongly primed defense response in Arabidopsis in SAR-induced plants is the *Psm*-triggered accumulation of the phytoalexin camalexin (Návarová et al., 2012). SAR is associated with enhanced expression of the camalexin biosynthetic genes *CYP71A13* and *PAD3* but not *CYP79B2*, and therefore provides partial activation of camalexin production. This implicates that fewer components would have to be induced in a future pathogen challenge to activate the whole response. Partial pre-activation of defense pathways might thus be a general mechanistic principle by which SAR-induced plants manage to accelerate defense responses when challenge-infected.
12. Varying environmental conditions can influence the magnitude and particular mechanistic aspects of the SAR response (Shah and Zeier, 2013). The quantitative differences we have observed for the transcriptional SAR responses of experiment 3 compared with the responses in experiments 1 and 2 were associated with higher overall leaf expression levels of major anthocyanin biosynthesis genes. Leaf anthocyanin accumulation is a characteristic response to unfavorable environmental conditions (Misyura et al., 2013). An important task for future SAR research will be to systematically investigate to what extent and how other environmental issues and stress parameters influence SAR establishment.

### Conflict of interest statement

The authors declare that the research was conducted in the absence of any commercial or financial relationships that could be construed as a potential conflict of interest.
